# Mapping the risk of avian influenza in wild birds in the US

**DOI:** 10.1186/1471-2334-10-187

**Published:** 2010-06-23

**Authors:** Trevon L Fuller, Sassan S Saatchi, Emily E Curd, Erin Toffelmier, Henri A Thomassen, Wolfgang Buermann, David F DeSante, Mark P Nott, James F Saracco, CJ Ralph, John D Alexander, John P Pollinger, Thomas B Smith

**Affiliations:** 1 Center for Tropical Research, Institute of the Environment, University of California, Los Angeles, La Kretz Hall, Suite 300, Box 951496, Los Angeles, CA 90095-1496, USA; 2 Radar Science Technical Group, Radar Science & Engineering Section, Jet Propulsion Laboratory, California Institute of Technology, 4800 Oak Grove Drive, Pasadena, CA 91109-8099, USA; 3 Department of Ecology and Evolutionary Biology, University of California, Los Angeles, 621 Charles E. Young Drive South, Los Angeles, CA 90095-1606, USA; 4 Department of Atmospheric and Oceanic Sciences, University of California, Los Angeles, Los Angeles, CA 90095-1565, USA; 5 The Institute for Bird Populations, P.O. Box 1346, Point Reyes Station, CA 94956-1346, USA; 6 U.S. Department of Agriculture, Forest Service, Pacific Southwest Research Station, Redwood Sciences Laboratory, 1700 Bayview Drive, Arcata, CA 95521, USA; 7 Klamath Bird Observatory, P.O. Box 758, Ashland, OR 97520, USA

## Abstract

**Background:**

Avian influenza virus (AIV) is an important public health issue because pandemic influenza viruses in people have contained genes from viruses that infect birds. The H5 and H7 AIV subtypes have periodically mutated from low pathogenicity to high pathogenicity form. Analysis of the geographic distribution of AIV can identify areas where reassortment events might occur and how high pathogenicity influenza might travel if it enters wild bird populations in the US. Modelling the number of AIV cases is important because the rate of co-infection with multiple AIV subtypes increases with the number of cases and co-infection is the source of reassortment events that give rise to new strains of influenza, which occurred before the 1968 pandemic. Aquatic birds in the orders Anseriformes and Charadriiformes have been recognized as reservoirs of AIV since the 1970s. However, little is known about influenza prevalence in terrestrial birds in the order Passeriformes. Since passerines share the same habitat as poultry, they may be more effective transmitters of the disease to humans than aquatic birds. We analyze 152 passerine species including the American Robin (*Turdus migratorius*) and Swainson's Thrush (*Catharus ustulatus*).

**Methods:**

We formulate a regression model to predict AIV cases throughout the US at the county scale as a function of 12 environmental variables, sampling effort, and proximity to other counties with influenza outbreaks. Our analysis did not distinguish between types of influenza, including low or highly pathogenic forms.

**Results:**

Analysis of 13,046 cloacal samples collected from 225 bird species in 41 US states between 2005 and 2008 indicates that the average prevalence of influenza in passerines is greater than the prevalence in eight other avian orders. Our regression model identifies the Great Plains and the Pacific Northwest as high-risk areas for AIV. Highly significant predictors of AIV include the amount of harvested cropland and the first day of the year when a county is snow free.

**Conclusions:**

Although the prevalence of influenza in waterfowl has long been appreciated, we show that 22 species of song birds and perching birds (order Passeriformes) are influenza reservoirs in the contiguous US.

## Background

There is a strong link between influenza in birds and human health because influenza epidemics in human populations occur when viruses that typically inhabit the avian gastrointestinal tract mutate or reassort, enabling them to cross the species barrier to infect people [1]. Mutations arise in avian influenza virus (hereafter "AIV") due to the high error rate of influenza RNA polymerase and the large population size and short generation time of the virus [2]. Reassortment is the exchange of RNA segments between distinct influenza viruses. When human influenza viruses and AIV reassort, they produce offspring virions that represent a mixture of the parental types' RNA and are infectious to humans in some cases [2-4]. For example, in 1968, one million people died in an influenza pandemic that resulted from the reassortment of an influenza virus from Ukrainian ducks and a virus that had circulated in people since 1957 [5-8].

Today, outbreaks of H5N1 influenza in Africa, Asia, Europe, and the Middle East further illustrate the human health implications of influenza in birds. (Influenza viruses are classified into "HA" and "NA" subtypes based on surface proteins.) People contract H5N1 by handling infected poultry or wild birds after which the virus binds to receptors in the pulmonary alveoli, causing pneumonia and death due to respiratory failure [9-11]. Since July 2003, there have been 436 human cases of H5N1 in the Eastern hemisphere with a 60% mortality rate [12-14]. In 75% of these cases, the infected people had contact with birds [10]. However, H5N1 has also evolved limited person-to-person transmission within human families [7,15-17]. Public health authorities are concerned that the evolution of wider human-to-human transmission could result in a H5N1 pandemic that could cause up to 142 million deaths at a cost of $US 4.4 trillion [14,18]. The ongoing human pandemic of H1N1 influenza, which has caused over 296,000 human cases and at least 15,921 deaths since mid-February 2009, contains genes from avian, human, and swine influenza viruses [12].

To date, influenza viruses have been isolated from 105 species of wild birds representing 26 families [16]. In birds, the H5 and H7 AIV subtypes have periodically mutated from a low pathogenicity form (hereafter "LPAI"), which is typically asymptomatic in wild birds, to a highly pathogenic form (hereafter "HPAI") that causes mortality rates of up to 100% in chickens [2,19-21]. (Our analysis did not distinguish between influenza subtypes or differentiate LPAI from HPAI.) HPAI also differs from LPAI in that the former has more amino acids adjacent to the hemagglutinin cleavage site, which allows it to replicate in a broader range of tissues [for details, see [2,22]]. Aside from poultry, no HPAI H5N1 has been detected to date in the US, though six LPAI H5N1 viruses have been detected in North America since 2004 [23]. AIV mutated from LP to HP form in poultry in the US in the 1920s, in 1984, and in 2004 [4,24-29]. Although none of these US outbreaks resulted in the infection of humans with HPAI, it is plausible that HPAI could reassort or mutate to become transmissible to people. As few as five amino acid changes can transform the HP influenza virus into an airborne form that is infectious to mammals [14]. In the event of an HPAI epizootic in migratory birds in the US, these species could spread HPAI across the country along migratory routes because ducks infected with HP H5 remain healthy enough to migrate [16,30]. Indeed, HPAI has already been detected in wild birds in Chad, China, Nigeria, and South Africa [30-32].

We analyze the geographic distribution of AIV in wild birds in the US with the goal of inferring where reassortment events might occur and how HPAI might travel if it enters wild bird populations. Our method for detecting the influenza virus in samples from passerines does not determine whether the virus is LPAI or HPAI (see below). However, 67% of our samples are from non-passerines and are known to be LPAI. Thus, this study assumes that most of our AIV-positive samples are LPAI. We model the geographic distribution of AIV to provide insights about how HPAI might spread if it is introduced to the US in the future. Since we cannot guarantee that the passerine samples are LPAI, when referring to samples that tested positive for influenza virus, we will use the term "AIV" rather than "LPAI". As noted in the Discussion, the characterization of the subtype and pathogenicity of AIVs isolated from passerines in the US remains an important area for future research.

Although the monitoring of HPAI viruses is important, another critical issue in AIV biosafety is the detection of H5 and H7 LPAI viruses. LPAI H7 has been transmitted directly to humans in the US in 1976, 2002, and 2003 [7,33]. These cases resulted in conjunctivitis, fever, and upper-respiratory tract symptoms of influenza-like illness, but no fatalities. LPAI H5 and H7 can mutate to HPAI relatively easily given the right environment (for example, poultry sheds). We refer the interested reader to Verdugo et al. [34], who have developed a model for detecting H5 and H7 LPAI in poultry and predicting when they will evolve to HPAI.

The aims of this research are to measure the prevalence of AIV in different species of wild birds in the US and to prioritize geographical regions for future influenza surveillance. Although aquatic birds in the orders Anseriformes and Charadriiformes have been recognized as reservoirs of AIV since the 1970s, much less is known about AIV prevalence in terrestrial birds in the order Passeriformes [16,35-38]. Examples of Anseriformes (ducks) that have high prevalence of influenza in the US include the Mallard (*Anas platyrhynchos*) and the Northern Pintail (*Anas acuta*) [39,40]. Shorebirds of the order Charadriiformes in which high influenza prevalence has been detected in the US include the Ruddy Turnstone (*Arenaria interpres*) and the Red Knot (*Calidris canutus*) [36,41]. Recent work detected high prevalence of influenza in passerines in China, including the Eurasian Tree Sparrow (*Passer montanus*) [42,43]. The present study is necessary in order to test the hypothesis that passerines are important reservoirs of AIV in the US. Further motivation for our study comes from the fact that public health agencies have limited funding to test wild birds for AIV. Thus, it is crucial that the establishment of surveillance sites should be as efficient as possible. For example, the number of sites that are monitored should be small but the sites should be located in counties that are most likely to have birds with AIV. We aim to test the hypothesis that environmental variables can be used to predict AIV cases in wild birds. Next, based on the relationship between AIV and environmental predictors, we attempt to identify the US counties most likely to be influenza hotspots for wild birds.

Our study makes the following contributions. First, guidelines formulated by the World Health Organization recognize the importance of epidemiological modelling using tools such as GIS for the control of AIV [44]. Nevertheless, most previous work on the geographic distribution of AIV has analyzed Asia and Africa [e.g. [35,38,45]]. To date, studies of AIV in wild birds in the US have focused on Alaska [39,46,47]. However, there may be overlooked hotspots of AIV in the contiguous US [48]. We contribute the first predictions about AIV cases in the contiguous US at the county scale. Second, we analyze new passerine samples from the Atlantic, Mississippi, and Pacific Flyways supplemented with existing samples from online databases to provide the first comprehensive assessment of AIV prevalence in US passerines. The main finding reported in this article is that the prevalence of influenza in passerines is greater than the prevalence in eight other avian orders. The implication of this finding for human health is that, along with poultry and waterfowl, passerines in the US are a potential vector for the transmission of AIV to people [16,49].

## Methods

### Influenza samples from wild birds

**Figure 1:**
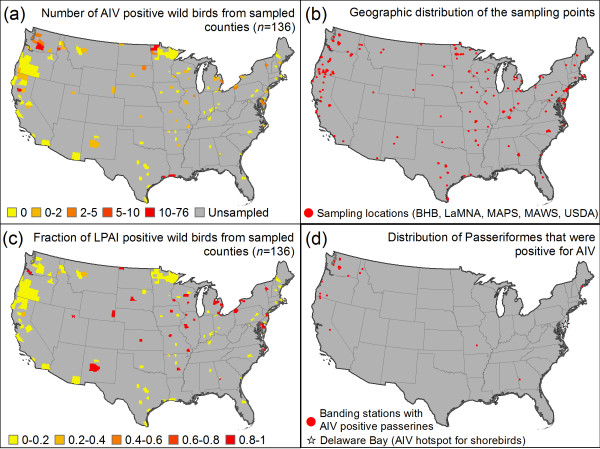
**Empirical data: AIV cases in the contiguous US**. (a) Number of positive wild birds in each of the sampled counties and county equivalents (*n *= 136). The data are partitioned into classes based on Jenks' natural breaks. (b) Locations of the bird banding stations where cloacal samples were collected as part of the LaMNA, MAPS, and MAWS banding networks. Also shown are the locations of geographic data from BioHealthBase (BHB) and the USDA. (c) Fraction of AIV positive samples. The upper Mississippi River basin and eastern Plains regions are hotspots based on the fraction of positives. (d) Distribution of AIV-positive samples from Passeriformes.

The data set comprised 13,046 samples from 136 counties or parishes in 41 US states (Figure 1, Additional file 1, Table S1, and Additional file 2, Table S1). The new samples included in this analysis comprise cloacal swabs collected from December 2005 to 2008 primarily during the Spring breeding season at banding stations that are part of the Monitoring Avian Productivity and Survival (MAPS) program, during the Fall as part of the Landbird Monitoring Network of the Americas (LaMNA), and during the Winter as part of the Monitoring Avian Winter Survival (MAWS) program in collaboration with UCLA's Center for Tropical Research [50,51]. Since it is routine for the samples collected by the banding network to be stored at room temperature for prolonged periods, we utilized 100% ethanol as a storage medium. Viral RNA was extracted using a commercial magnetic bead kit (Ambion MagMAX viral RNA isolation kit). The vRNA was then converted to cDNA in a real-time reverse transcription PCR reaction using an Ambion AgPath-ID one-step PCR kit and run on a 7900 HT Fast Real-Time PCR System. PCR primers targeted a conserved region of the Matrix 1 gene: 5-GAR ATC GCG CAG ARA CTT GA-3 and 5-CAC TGG GCA CGG TGA GC-3 are forward and reverse primers, respectively. For analysis of the sensitivity of the MagMAX and AgPath-ID kits, see [47]. We used High Resolution Melt Analysis to identify putative positives based on melt temperature and then confirmed amplicon length (143 bp) on a 3% agarose gel. Viral cDNA was purified using a Zymoclean DNA gel recovery kit. We used the same PCR primers in BigDye Terminator sequencing reactions and then products were run on an Applied Biosystems 3730 system. Chromatograms were visualized in Geneious and then confirmed as AIV through a BLAST search of the NCBI database. The field and laboratory methods utilized in this study were approved by an Institutional Review Board at the University of California, Los Angeles. AIV-positive samples have been deposited in GenBank under Accession Numbers HM355888 to HM355917.

Since our samples were cloacal swabs with no measurable volume, we could not estimate the viral load of influenza in passerines defined as the number of copies of the virus per unit of body fluid. We can nevertheless infer that our copy number is very low based on cycle of threshold (Ct) values, which is the cycle at which the samples began to amplify. Since the Ct values were fairly high, we estimate that our copy number is uniformly low across the passerine samples. (Ct values >35 are typically interpreted as indicating the absence of influenza in passerines [52].)

### Environmental variables

We investigated the association between AIV cases in wild birds in the contiguous US and 12 predictor variables, which measured agricultural and commercial activity as well as climate (Table 1). The predictor variables are all publicly available. First, we analyzed measures of agricultural activity because rice crop production is correlated with H5N1 outbreaks in Southeast Asia [38]. We included the amount of harvested cropland in the county in units of hectares, the mean size of farms in the county, and the % of the county in cropland [53]. We also examined the % of family-owned farms in the county because the use of employees who were not family members was significant for explaining AIV outbreaks in Virginia in 2002 [26]. In addition, we analyzed the density of roads in each county that connect major population centers and the human population density (as a surrogate measure of trade activity) because proximity to trade routes such as highways has been hypothesized to explain the spread of H5N1 from Asia to Europe in 2005 and proximity to roads was found to be a significant variable for explaining outbreaks of HPAI H5N1 in poultry in Romania in 2005 [54,55]. The density of poultry in the county was included in the analysis to test the hypothesis that poultry spread AIV to wild birds [56].

**Table 1:** Environmental variables used to predict AIV in wild birds in the US.

Variable	Source	Manipulation
Binary variable: 1 if the county ever froze, 0 otherwise	ftp://sidads.colorado.edu/pub/DATASETS/NOAA/G02156/metadata/	Calculated from freeze and thaw date
Freeze date	ftp://sidads.colorado.edu/pub/DATASETS/NOAA/G02156/metadata/	Calculated as the first day of the year when more than half of the county was frozen
Harvested cropland (ha)	http://www.nationalatlas.gov/atlasftp.html	None
Human population density	http://www.nationalatlas.gov/atlasftp.html	The total human population was divided by the area of the county
Mean farm size (ha)	http://www.nationalatlas.gov/atlasftp.html	None
Minimum temperature (°C)	http://www.prism.oregonstate.edu/products/matrix.phtml?year0=2000	Calculated as the average minimum temperature of all of the 800 m pixels in the county
Percent family owned farms	http://www.nationalatlas.gov/atlasftp.html	None
Percent of county in cropland	http://www.nationalatlas.gov/atlasftp.html	None
Poultry density	http://www.fao.org/geonetwork/srv/en/resources.get?id=12720&fname=glbpo25cor.zip&access=private	The data were aggregated to the county scale and the total number of poultry was divided by the area of the county
Road density	http://www.nationalatlas.gov/atlasftp.html	Calculated as the number roads in each county that connect major population centers
Thaw date (°C)	ftp://sidads.colorado.edu/pub/DATASETS/NOAA/G02156/metadata/	Calculated as the first day of the year when more than half of the county was not frozen
Total annual precipitation (mm)	http://www.prism.oregonstate.edu/products/matrix.phtml?year0=2000	Calculated as the average precipitation of all of the 800 m pixels in the county

We incorporated total annual precipitation and minimum temperature data interpolated from weather stations using the Parameter-elevation Relationships on Independent Slopes Model (PRISM) method, which accounts for physiographic features such as terrain barriers that result in rain shadows and is considered the most accurate representation of US climate patterns [57]. Previous work has posited a relationship between temperature and influenza cases. For example, the 1918 pandemic coincided with an unusually hot winter in eastern North America and north central Asia caused by one of the strongest El Niño/Southern Oscillation (ENSO) events of the twentieth century [58]. We hypothesized that precipitation and minimum temperature might affect influenza prevalence in wild birds because AIV can be transmitted to birds in a moist environment and cold weather affects bird dispersal [59], which may influence the spread of the virus [60,61]. (For these and the following meteorological variables, we used the average value of the variable from 2006 to 2008 because our AIV samples came from December 2005 to 2008). Finally, we examined: (i) a binary variable set to one if the majority of the land in the county was ever covered by snow/ice and set to zero otherwise, (ii) the day of the year when the majority of the land in the county was first covered by snow/ice, and (iii) the day of the year when the majority of the land in the county was first free of snow/ice, all of which were inferred from satellite radar [62]. We included these variables related to freeze and thaw date because the timing of spring migration depends on the first day of thaw and fall migration depends on the first day of freeze. These variables may indirectly explain AIV spread by affecting bird dispersal [63]. Variables were iteratively removed from the spatial regression model via backward elimination until only variables with a *t*-statistic in the 95^th ^percentile remained [64]. Forward selection gave the same result.

### Spatial regression model

The statistical model represents the number of AIV cases per county as a Poisson-distributed random variable, which is appropriate for analyzing disease cases in which some geographic units have many cases but most units have few or no cases [65]. In the model, the number of AIV cases per county depends on the environmental variables as well as spatial proximity to other counties with AIV cases. In particular, we utilized a spatial regression model to account for autocorrelation in AIV cases among counties that are close together geographically. Failure to address autocorrelation results in underestimation of the degrees of freedom of the data, which decreases the standard errors of the parameter estimates in a regression model [66]. Thus, ignoring autocorrelation may lead to the erroneous conclusion that a variable is significant for explaining AIV cases when the variable is in fact non-significant. The model incorporates spatial autocorrelation by constructing a semivariogram and also accounts for differences in sampling effort among counties [67]. After the model was fitted to the 136 counties for which we had AIV samples, we predicted the number of AIV cases in the other 2973 US counties by applying the model to the unsampled counties. We utilized a generalized linear mixed model, which is a form of kriging with a semivariogram, rather than a conditional autoregressive model because the latter cannot readily be extended to non-Gaussian data [67]. The Poisson distribution provided a better fit to our data on flu cases than the Gaussian distribution (see Additional file 3). The accuracy of the spatial regression was assessed using two measures. First, we calculated the generalized chi-squared statistic. If a regression model is accurate, then the generalized chi-squared statistic divided by the degrees of freedom of the data should be close to one [67,68]. The model provided a good fit to the data insofar as the generalized chi-squared statistic divided by the degrees of freedom was 0.9. Collinearity among the regressors was also acceptably low (variance inflation factors <1.75) [68]. Second, we used a leave-one-out procedure that fitted the model to the data from 135 counties and then measured the model's accuracy on the remaining county. When the procedure was repeated 136 times, the root mean squared error (r.m.s.e.) was 6.33 AIV cases per county. This r.m.s.e. is acceptable based on the rule of thumb that the r.m.s.e. should be no greater than one-quarter of the range [69]. (For our data set, the range was 76.) Further details of the statistical model can be found in Additional file 3.

## Results

**Figure 2:**
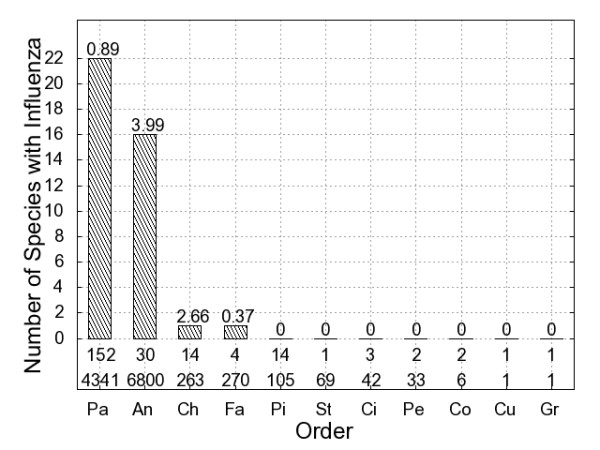
**Influenza prevalence among avian orders**. Each bar represents one order of birds in the contiguous US. The height of the bar indicates that number of species belonging to the order that tested positive for AIV. The number above each bar is the % of cloacal samples that were AIV-positive. An = Anseriformes, Ch = Charadriiformes, Ci = Ciconiiformes, Co = Columbiformes, Cu = Cuculiformes, Fa = Falconiformes, Gr = Gruiformes, Pa = Passeriformes, Pe = Pelecaniformes, Pi = Piciformes, and St = Strigiformes. The first number below the bar is the number of species that we tested from that order. The second number is the number of samples that we tested from all of the species belonging to the order (see Additional file 2, Table S1 for details).

Among the eight avian orders analyzed here, Passeriformes had the largest number of species in which AIV was detected (22 species) followed by ducks (order Anseriformes, 16 species) and shorebirds (order Charadriiformes, 1 species). We tested 4,341 samples from passerine birds, of which 0.89% were AIV-positive (Figure 2). Table 2 lists the top five species in terms of AIV-prevalence for each avian order.

**Table 2:** AIV prevalence among avian orders.

AIV-positive species	Common name	Prevalence (%)
**Anseriformes**		
*Cygnus olor*	Mute Swan	100
*Anas rubripes*	American Black Duck	50
*Aythya Americana*	Redhead	33.3
*Anas carolinensis*	Green-winged Teal	7.36
*Anas platyrhynchos*	Mallard	5.9
**Charadriiformes**		
*Arenaris interpres*	Ruddy Turnstone	16.3
**Falconiformes**		
*Haliaeetus leucephalus*	Bald Eagle	1.23
**Passeriformes**		
*Regulus satrapa*	Golden-crowned Kinglet	50
*Passerella iliaca*	Fox Sparrow	10
*Piranga ludoviciana*	Western Tanager	9.09
*Seiurus noveboracensis*	Northern Waterthrush	9.09
*Carpodacus cassinii*	Cassin's Finch	8.33

Two orders (Charadriiformes and Falconiformes) had only one AIV-positive species. For orders in which influenza was detected in at least five species, the top five species in terms of prevalence are listed below.

### Influenza prevalence in Passeriformes

We evaluated the hypothesis that AIV was equally prevalent among 11 avian orders by testing the null hypothesis that AIV prevalence was the same among the orders. The null hypothesis was rejected, indicating that AIV is more common in some orders than in others (Kruskal-Wallis *KW *= 124, *df *= 10, *p *< 0.0001). Next, we ranked the orders based on AIV prevalence. The number of passerine species that tested positive for AIV (*n *= 22) was greater than the number of positive species detected in ten other orders, including waterfowl. However, our sampling was also biased towards passerines. To address this, we calculated fraction of AIV-positive samples from each order. This corrects for the fact that we had more samples from some orders than from others. The fraction of samples from passerines that tested positive for AIV was greater than the prevalence in eight other orders of birds in the contiguous US (Figure 2). We note, however, that for some of these eight orders the number of species and samples represented in our database is small. Nevertheless, AIV prevalence in Passeriformes was greater than in Falconiformes, an order in which we tested four species and 270 samples, and Piciformes, and order in which we tested 14 species and 105 samples.

### Environmental predictors of influenza prevalence in the sampled counties

For the remainder of the analysis, we pooled the samples from all 11 orders so as to increase the geographic region represented by data. Significant predictors of the number of AIV cases per county in wild birds were thaw date, the % of harvested cropland in the county, and minimum temperature (Table 3). We interpret each of these variables in turn beginning with thaw date. Freeze/thaw dynamics have previously been hypothesized to explain the prevalence of AIV in ducks in Europe (Andrew Dobson, Princeton University, personal communication, 2009). We posit that thaw date affects influenza in wild birds in the US according to the following mechanism. Waterfowl have large populations with high annual turnover rates, so that a large fraction of the population is immunologically naïve each year [70]. In particular, hatchlings are susceptible to infection from adults via fecal-oral transmission at breeding grounds, which have densities of up to 210 birds/m^2 ^[20,37]. At these sites, adults shed the virus into the water in feces and the young are infected by ingesting the water [4]. Prevalence of AIV in gulls in the US is typically highest in spring and in summer [71]. The sign of the coefficient for thaw date is negative, indicating that if the thaw date occurs later in the year, then the number of cases of AIV is predicted to decrease [Table 3, for details see [67]]. We conjecture that thaw date explains AIV cases because if a site thaws earlier, then waterfowl can occupy the site sooner, and there are more opportunities for adults to infect juveniles than if the site were to become free of snow and ice later in the year. In our data set, the prevalence of influenza among hatchling year birds is significantly greater than in second-year or adult birds (*x*_1_^2 ^= 64.87, one-sided *p *= 4 × 10^-16^). That finding is compatible with the hypothesis that a high fraction of hatchlings are infected by adults at the breeding grounds.

**Table 3:** Effect of the environmental variables on AIV cases in wild birds in the contiguous US, 2005-2008.

Effect	**Est**.	**Std. Err**.	*t*-value (df = 132)	*p*
Intercept	-3.279	0.58	-5.65	<.0001
First day of the year free of ice or snow Range: 0 (snow-free) to day of the year 150	-0.0282	0.0116	-2.43	0.0164
Minimum temperature Range: -7.15 to 19.67°C	-0.2143	0.0734	-2.92	0.0041
Percent harvested cropland per county: Range: 0-100	0.0433	0.00951	4.56	<.0001

The amount of harvested cropland per county was very highly significant for explaining AIV cases. This result is consistent with previous analyses that showed a significant effect of agricultural production on AIV cases in Southeast Asia [38,72]. We hypothesize that agricultural activity results in increased AIV prevalence because it reduces the amount of natural habitat available to avian migrants. For example, the conversion of wetlands to farms may create bottlenecks at stopover sites along migratory corridors, concentrating waterfowl into high densities, such as the congregations of Teal and Snow Geese at Kesterson National Wildlife Refuge in California's Central Valley during migration. The resulting crowding and intermingling of different species is thought to increase the probability that a bird will be infected with influenza [22].

Minimum temperature also emerged as significant for explaining AIV cases. The sign of the coefficient that represents the effect of minimum temperature was estimated to be negative, meaning that if temperature increases, then the number of AIV cases is predicted to decrease (Table 3). This finding is compatible with the ecology of AIV, which is known to survive longer outside the host in cold conditions [2,73]. During the 1984 Pennsylvania outbreak of H5N2, the virus survived in barns for as long as 105 days during cool weather [28]. In addition, winters are long and cold at Qinghai Lake, China, which was the site of an outbreak of HPAI in wild birds in 2005 [32]. It has also been conjectured that cold snaps explain the spread of HPAI by wild birds in Europe insofar as cold weather events prompt the dispersal of infected birds, resulting in the spread of the virus [16,63,74,75]. For example, in 2006, mute swans that typically winter on the Black Sea became infected when bad weather forced them to leave. This led to the discovery of infected mute swans in Azerbaijan, Georgia, Iran, Kazakhstan, and 20 European countries [31]. Additionally, frost is one of the stress factors associated with dead wild birds infected with HPAI [16].

### Influenza predictions for the unsampled counties

**Figure 3:**
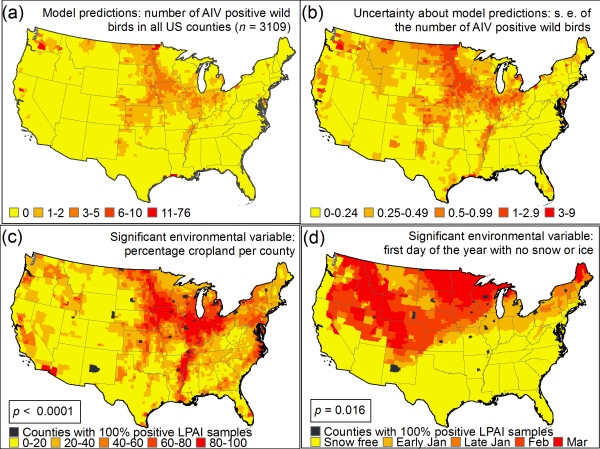
**Predictions of the spatial model**. (a) Predictions for AIV cases in sampled and unsampled counties (*n *= 3109). R.m.s.e.: 6.33 cases per county; (b) Uncertainty associated with the model predictions in (a): standard error of the number of predicted cases of AIV in wild birds; (c) An important environmental variable for explaining AIV cases: % cropland per county; (d) Another important environmental variable: first day of the year with no snow or ice. Most counties with a high % of AIV positive-wild birds have a high % crop of cropland and cold climate measured as snow or ice-cover in January. This is compatible with the observation that the AI virus can survive outside of the host for a longer time period in a cold environment.

**Figure 4:**
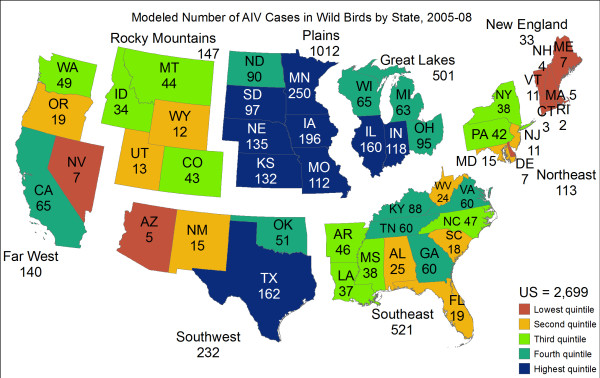
**Modelled number of AIV cases in wild birds per US state and region, 2006-2008**. States are colored based on quintiles so that states that are in the top 20% with respect to the number of AIV cases are colored dark blue and states in the bottom 20% are red-orange. The symbology is adapted from a map of US GDP [84].

The analysis at the county scale identifies the Mississippi River basin as a hotspot for AIV cases in the contiguous US (Figure 3(a)), which may be because wetlands in the basin with shallow pools of water are conducive to the transmission of the virus [37]. The Pacific Northwest is also classified as a hotspot, though this may be due in part to the fact that many of our samples of passerines came from this region (Figure 1(b), Additional file 4, Figure S1). At the state scale, Minnesota is predicted to have the most cases of AIV in the contiguous US (Figure 4). This is not surprising since AIV is known to have been introduced to turkey farms in Minnesota by wild birds 135 times since 1968 [76]. At the county scale, there is a pronounced north-to-south gradient in the predicted number of AIV cases in the contiguous US (Figure 3(a)). This finding is compatible with the theory that the virus persists better in the colder environments of the northern US than the warmer environment of southern US states. Finally, the model predicts a large number of AIV cases in the Corn Belt of the Central US (Figure 3(a)). This area is known to be a major migratory flyway for ducks and also has intensive agricultural production [53], which is a significant risk factor for AIV according to our model. If we reformulate the regression model to estimate the probability of AIV occurrence rather than estimating the number of cases of AIV, the spatial pattern of AIV in the contiguous US remains qualitatively similar (Additional file 5, Figure S1).

## Discussion

### Implications for influenza surveillance and control in wild birds

Although the role of ducks and wading birds as influenza reservoirs has long been appreciated, our analysis shows that land birds constitute an important natural host of the influenza virus in the US. Analysis of 225 avian species indicates that influenza prevalence is higher in passerines than in eight other orders of birds in the contiguous US. Thus, the implication of this study for surveillance is that passerines should be monitored as a potential vector for transmitting AIV to humans, in addition to water birds and domesticated birds.

Since vaccinating against or stamping out AIV in all wild birds would be impossible, it is important to prioritize populations for such management activities [16]. Our model predicts that the risk of AIV outbreaks in wild birds is highest in California, the Great Plains, Minnesota, Texas, and Washington (Figures 3 and 4). The Plains region is predicted to have the highest number of AIV cases due to its significant agricultural production and cold winter temperatures, which allow AIV to persist outside the host in environmental reservoirs (Figure 4). Our prediction that there will be an AIV hotspot in the Pacific Northwest is driven by our samples from passerine birds because almost all of the positive samples from Passeriformes came from this region. The Passeriformes with the highest AIV-prevalence was the Golden-crowned Kinglet (*Regulus satrapa*), but this result should be interpreted cautiously since we had few samples for this species (Table 2, Additional file 2, Table S1). The passerine bird that showed the highest prevalence among the species for which we had a large number of samples was Swainson's Thrush (*Catharus ustulatus*). Swainson's Thrush is a Neotropical migrant whose breeding sites include remote areas of the Pacific Northwest that are isolated from human settlements or farms [57]. The fact that we detected high AIV-prevalence in Swainson's Thrush supports the hypothesis that passerines birds constitute a reservoir of AIV even without the spillover of influenza from domestic birds to wild birds at farms in the breeding range. However, further work is needed to investigate the possible exposure of Swainson's Thrush to poultry in its winter range.

Conversely, the Mississippi Flyway in the Plains region had only one AIV-positive sample each from song birds and shorebirds, so our prediction of an AIV hotspot in this part of the country is determined primarily by influenza-positive ducks (Anseriformes). The Anseriformes species with the highest AIV prevalence was the Mute Swan (*Cygnus olor*), which had 100% prevalence in our data set (Table 1). However, this prevalence may not be representative of natural populations insofar as our sample size for this species was small. Among the well-sampled Anseriformes species, AIV-prevalence was high in the Mallard (*Anas platyrhynchos*) and the Green-winged Teal (*Anas carolinensis*) (Additional file 2, Table S1). This result is compatible with previous studies that have detected high prevalence in both of these duck species [reviewed in [72]].

In addition to estimating the mean number of AIV cases in wild birds per county, we predict the standard error of the number of cases (Figure 3(b)). If the standard error for a particular county is large, then there is a great deal of uncertainty about AIV cases in the county. To reduce this uncertainty, counties with large standard errors, such as those in central Washington and central Montana, can be targeted for increased surveillance.

### Human health implications of influenza in wild birds

Previous spatial models have predicted the occurrence and non-occurrence of AIV [38,45]. The novel contribution of our model is the prediction of the number of cases of influenza in wild birds. Understanding the number of AIV cases in a county, rather than the occurrence or non-occurrence of the virus, is important because the rate of co-infection with multiple influenza viruses increases with the number of AIV cases [2]. Co-infection is the source of reassortment events that give rise to new pandemic strains of AIV; indeed, such an event preceded the 1918 influenza pandemic that killed 50-100 million people [2,3,5-8,77,78]. Since the number of AIV cases is predicted to be highest in the Great Plains and the Pacific Northwest, we predict that these two geographical regions will also have a concomitantly higher risk of co-infection and reassortment. Thus, the threat to human health due to the evolution of HPAI through reassortment is greatest in those two parts of the country. As a consequence, implementing biosecurity practices for the control of HPAI is especially crucial in those two areas [79].

Efforts undertaken by US health departments to plan for AIV since 2005 are thought to have facilitated the response of federal, state, and local agencies to the novel H1N1 epidemic during the Spring of 2009 [80]. Thus, modelling the distribution of AIV in the US and allocating health care resources based on the predictions of such models may contribute to an improved technological infrastructure for responding to future epizootics and epidemics of influenza as well as other public health crises.

### Shortcomings of the analysis and areas for future research

Among the limitations of the analysis is that we did not construct separate models for AIV in different species of wild birds. However, this shortcoming may not be severe because H5 AIV does not show species-specific differences in North America [16,23]. Moreover, like our analysis, epidemiological models often analyze the distribution of the pathogen rather than that of the host species [e.g. [81]]. Another limitation of the analysis is that our viral storage and detection methodology does not permit the characterization of the HA or NA subtype of a sample that tests positive for influenza. This is a shortcoming because it may be more important to map the distribution of subtypes that are highly virulent in mammals such as H5 or H7. Our models are based on the detection of influenza A through matrix gene detection rather than the analysis of the H5 and H7 subtypes. Thus, if these two subtypes have unique geographic distributions because they are only transmitted by particular species of wild birds, then our modelling approach might fail to capture this. However, wild birds in the US exhibit a high rate of turnover in serotypes according to a roughly 2-year cycle [2,16,36,61,82]. Thus, modelling the geographic distributions of the H5 or H7 subtypes would require detailed annual surveys. Although such surveys have been conducted for Delaware Bay [49], such data are not available for sites that represent broad geographic sampling of the contiguous US.

In the Eastern hemisphere, cases of H5N1 AIV in people typically increase in cooler months [9]. We found that cool temperatures are also a good predictor of AIV cases in wild birds. Influenza dynamics in wild birds in the US appear to depend on climatic variables rather than a fixed annual cycle because the relationship between AIV cases and day of the year is weak (Pearson's = 0.17). A hypothesis that emerges from this study is that H5N1 may be more prevalent in humans during cool months because both the prevalence of the virus in birds and the rate of avian-to-human transmission are higher in cool weather. Future field studies could assess the evidence in support of this hypothesis by testing for influenza in birds and people simultaneously in the same geographic region during cool weather. If the hypothesis is not confirmed, a possible alternative explanation for the increase in human cases of influenza during cool weather is that people spend more time indoors during the cool winter months and thus have greater exposure to infected individuals [3].

The recent H1N1 pandemic has demonstrated the public health significance of reassortment events between avian and swine influenza viruses. Such reassortment might be expected to be more frequent in geographic regions where (i) swine production is intensive and (ii) there is also high prevalence of influenza in wild birds. Regions that score high for both (i) and (ii) could have a greater likelihood of influenza reassortment events in livestock or wildlife hosts of the influenza virus. For example, results indicate that the Mississippi Flyway in the central US has significant swine production along with a significant number of cases of AIV in ducks (Additional file 6, Figure S1). However, the risk of an influenza epidemic in humans may depend on other parameters, such as contact rates among people, wild birds, poultry, and swine, and the transmission efficiency of the virus. The available data on the exposure of humans to AIV is extremely limited and difficult to interpret due to a lack of standardized methods for serological testing [reviewed in [7]]. We consider the improvement of such data an important avenue for future work and refer the interested reader to Moffett et al. [83] for the incorporation of contact rates and transmission efficiencies into a model of epidemiological risk.

## Conclusions

The main conclusion of this research is that land birds (Passeriformes) constitute an important natural reservoir of influenza in the contiguous US. The importance of this finding is that since passerine species are common in urban habitats they could readily transmit highly pathogenic influenza to people in the event that a highly pathogenic subtype evolves through mutation or through reassortment in a bird that is co-infected with distinct influenza viruses. Aquatic birds are typically referred to as the most important avian vector of influenza. However, since passerines occupy the same habitat as poultry and there have already been outbreaks of HPAI in US poultry, passerines may be more effective at transmitting HPAI to people than aquatic birds. The geographical regions with the highest risk of influenza in wild birds are the Great Plains and the Pacific Northwest; the threat to human health due to reassortment events that produce pandemic influenza subtypes is also greatest in these two areas.

## Competing interests

The authors declare that they have no competing interests.

## Authors' contributions

TF designed the study's analytical methods and drafted the manuscript. SS helped design the analytic methods, including the preparation of the satellite data described in the Methods. EC supervised the first year of laboratory activities, helping to design laboratory techniques for influenza testing, and wrote a FileMaker Pro database to summarize the samples. ET supervised the second year of laboratory activities including flu testing and the cataloguing of more than 10,000 cloacal samples. HT helped design the analytic aspects of the study and carried out a review of the literature about flu in wild birds. WB helped design the study's analytic strategy including the selection of the type of remote-sensed images to be used in the spatial model and selected the spatial scale of these images. DD designed the study's field activities including the sampling of passerine birds as part of the MAPS bird banding network. PN reviewed and revised a preliminary draft of the manuscript, provided guidance about the study's analytical approach, and helped design sampling activities as part of the MAPS network. JM supervised field activities as part of the MAWS bird banding network and designed Figure 2 of the manuscript. CJR supervised field activities in the LaMNA bird banding network, which provided flu samples during the migratory period. JA helped supervised the LaMNA bird banding stations and revised a draft of the article. JP directed laboratory activities during both years of flu testing and helped draft the Methods section of the manuscript. TBS helped design the analytic aspect of the study and helped draft the manuscript. All authors read and approved the final manuscript.

## Pre-publication history

The pre-publication history for this paper can be accessed here:

http://www.biomedcentral.com/1471-2334/10/187/prepub

## Supplementary Material

Additional file 1 **Influenza samples from wild birds used in the study**. This file provides a detailed description of the geographical study region and lists the online databases from which we obtained samples in addition to the samples tested at the UCLA Center for Tropical Research. Click here for file

Additional file 2 **Description of the samples by species**. The table in this file reports the prevalence of flu in the 225 avian species analyzed in this study, which represent 11 orders of birds. Click here for file

Additional file 3 **Formulation of the spatial regression model**. This file explains how we constructed the semivariogram in the spatial regression model, provides a mathematical formulation of the model, and explains how we fitted the model. Click here for file

Additional file 4 **Geographic locations of AIV-positive samples in the contiguous US (*n *= 325)**. This file contains a map showing the bird banding stations where wild birds tested positive for AIV. Click here for file

Additional file 5 **Probability of AIV occurrence in US counties or county equivalents**. This file consists of a map constructed by modifying the spatial model to generate probabilistic predictions about the influenza in wild birds, which are restricted to being between zero and one, rather than estimates of the number of influenza cases, which range from zero cases to 76 cases per county. Click here for file

Additional File 6 **Hotspots of swine production and AIV cases in wild birds in the contiguous US**. This file shows the overlap between areas with intensive swine production in the US and areas in which we predict high prevalence of AIV in wild birds. Reassortment between avian and swine influenza viruses may be more common in such areas. Click here for file

## References

[B1] Bush RM Tibayrenc M Hoboken Influenza evolution Encyclopedia of Infectious Diseases: Modern Methodologies 2007 New Jersey, USA: John Wiley & Sons 199 214 full_text

[B2] Clark L Hall J Avian influenza in wild birds: status as reservoirs, and risks to humans and agriculture Ornithological Monographs 2006 60 3 29 10.1642/0078-6594(2006)60[3:AIIWBS]2.0.CO;2

[B3] Webster RG Bean WJ Gorman OT Chambers TM Kawaoka Y Evolution and ecology of influenza A viruses Microbiological Reviews 1992 56 152 179 1579108 10.1128/mr.56.1.152-179.1992 PMC372859

[B4] Bean WJ Kawaoka Y Wood JM Pearson JE Webster RG Characterization of virulent and avirulent A/Chicken/Pennsylvania/81 influenza A viruses: potential role of defective interfering RNAs in nature Journal of Virology 1985 54 151 160 3973976 10.1128/jvi.54.1.151-160.1985 PMC254772

[B5] McLeod A Swayne DE The economics of avian influenza Avian Influenza 2008 Ames, Iowa: Blackwell Publishing 537 560 full_text

[B6] Steel J Palese P Klenk H-D, Mastrosovich MN, Stech J The 1918 influenza pandemic: lessons from the past raise questions for the future Avian Influenza Monographs in Virology 2008 27 Basel: Karger 272 286

[B7] Cox NJ Uyeki TM Swayne DE Public health implications of avian influenza viruses Avian Influenza 2008 Ames, Iowa: Blackwell 453 484 full_text

[B8] Kilbourne ED Influenza pandemics of the 20th century Emerging Infectious Diseases 2006 12 9 14 16494710 10.3201/eid1201.051254 PMC3291411

[B9] Abdel-Ghafar AN Chotpitayasunondh T Gao Z Hayden FG Hien ND de Jong MD Naghdaliyev A Peiris M Shindo N Soeroso S Uyeki TM Avian influenza A (H5N1) virus infection in humans New England Journal of Medicine 2008 358 261 273 10.1056/NEJMra0707279 18199865

[B10] Gambotto A Barratt-Boyes SM de Jong MD Neumann G Kawaoka Y Human infection with highly pathogenic H5N1 influenza virus Lancet 2008 371 1464 1475 10.1016/S0140-6736(08)60627-3 18440429

[B11] Mastrosovich MN Gambaryan AS Klenk HD Klenk H-D, Mastrosovich MN, Stech J Receptor specificity of influenza viruses and its alteration during interspecies transmission Avian Influenza Monographs in Virology 2008 27 Basel: Karger 134 155

[B12] Neumann G Noda T Kawaoka Y Emergence and pandemic potential of swine-origin H1N1 influenza virus Nature 2009 459 931 939 http://www.who.int/csr/disease/swineflu/updates/en/index.html 10.1038/nature08157 19525932 PMC2873852

[B13] Feare CJ The role of wild birds in the spread of HPAI H5N1 Avian Diseases 2007 51 440 447 10.1637/7575-040106R1.1 17494603

[B14] DiNapoli JM Nayak B Yang LJ Finneyfrock BW Cook A Andersen H Torres-Velez F Murphy BR Samal SK Collins PL Bukreyev A Newcastle disease virus-vectored vaccines expressing the hemagglutinin or neuraminidase protein of H5N1 highly pathogenic avian influenza virus protect against virus challenge in monkeys Journal of Virology 2010 84 1489 1503 10.1128/JVI.01946-09 19923177 PMC2812327

[B15] Zou SM Potential impact of pandemic influenza on blood safety and availability Transfusion Medicine Reviews 2006 20 181 189 10.1016/j.tmrv.2006.03.001 16787826 PMC7134961

[B16] Artois M Bicout D Doctrinal D Fouchier R Gavier-Widen D Globig A Hagemeijer W Mundkur T Munster V Olsen B Outbreaks of highly pathogenic avian influenza in Europe: the risks associated with wild birds Revue Scientifique et Technique-Office International des Epizooties 2009 28 69 92 10.20506/rst.28.1.1854 19618620

[B17] Neumann G Chen H Gao GF Shu YL Kawaoka Y H5N1 influenza viruses: outbreaks and biological properties Cell Research 2010 20 51 61 10.1038/cr.2009.124 19884910 PMC2981148

[B18] McKibbin WJ Sidorenki AA Global Macroeconomic Consequences of Pandemic Influenza 2006 Sydney, Australia: Lowry Institute for International Policy

[B19] Tanimura N Tsukamoto K Okamatsu M Mase M Imada T Nakamura K Kubo M Yamaguchi S Irishio W Hayashi M Pathology of fatal highly pathogenic H5N1 avian influenza virus infection in large-billed crows (*Corvus macrorhynchos*) during the 2004 outbreak in Japan Veterinary Pathology 2006 43 500 509 10.1354/vp.43-4-500 16846992

[B20] Munster VJ Fouchier RAM Avian influenza virus: Of virus and bird ecology Vaccine 2009 27 6340 6344 10.1016/j.vaccine.2009.02.082 19840670

[B21] Fouchier RAM Munster VJ Epidemiology of low pathogenic avian influenza viruses in wild birds Revue Scientifique et Technique (Office International des Epizooties) 2009 28 49 58 10.20506/rst.28.1.1863 19618618

[B22] Osterhaus ADME Munster VJ Fouchier RAM Klenk H-D, Matrosovich MN, Stech J Epidemiology of avian influenza Avian Influenza Monographs in Virology 2008 27 Basel: Karger 1 10

[B23] Spackman E Swayne DE Suarez DL Senne DA Pedersen JC Killian ML Pasick J Handel K Somanathan Pillai SP Lee CW Characterization of low-pathogenicity H5N1 avian influenza viruses from North America Journal of Virology 2007 81 11612 11619 10.1128/JVI.01368-07 17728231 PMC2168782

[B24] Beaudette FR Hudson CB Saxe AH An outbreak of fowl plague in New Jersey in 1929 Journal of Agricultural Research 1934 49 83 92

[B25] Halvorson DA Prevention and management of avian influenza outbreaks: experiences from the United States of America Revue Scientifique et Technique-Office International des Epizooties 2009 28 359 369 10.20506/rst.28.1.1866 19618639

[B26] McQuiston JH Garber LP Porter-Spalding BA Hahn JW Pierson FW Wainwright SH Senne DA Brignole TJ Akey BL Holt TJ Evaluation of risk factors for the spread of low pathogenicity H7N2 avian influenza virus among commercial poultry farms Journal of the American Veterinary Medical Association 2005 226 767 772 10.2460/javma.2005.226.767 15776951

[B27] van der Goot JA Koch G de Jong MCM van Boven M Transmission dynamics of low- and high-pathogenicity A/Chicken/Pennsylvania/83 avian influenza viruses Avian Diseases 2003 47 939 941 10.1637/0005-2086-47.s3.939 14575091

[B28] Swayne DE Swayne DE High pathogenicity avian influenza in the Americas Avian Influenza 2008 Ames, Iowa: Blackwell 191 216 full_text

[B29] Lee CW Swayne DE Linares JA Senne DA Suarez DL H5N2 avian influenza outbreak in Texas in 2004: The first highly pathogenic strain in the United States in 20 years? Journal of Virology 2005 79 11412 11421 10.1128/JVI.79.17.11412-11421.2005 16103192 PMC1193578

[B30] Gaidet N Cattoli G Hammoumi S Newman SH Hagemeijer W Takekawa JY Cappelle J Dodman T Joannis T Gil P Evidence of infection by H5N2 highly pathogenic avian influenza viruses in healthy wild waterfowl PLoS Pathogens 2008 4 e1000127 10.1371/journal.ppat.1000127 18704172 PMC2503949

[B31] Alexander DJ Brown IH History of highly pathogenic avian influenza Revue Scientifique et Technique-Office International des Epizooties 2009 28 19 38 10.20506/rst.28.1.1856 19618616

[B32] Prousser DJ Takekawa JY Newman SH Yan B Douglas DC Hou Y Xing Z Zhang D Li T Li Y Satellite-marked waterfowl reveal migratory connection between H5N1 outbreak areas in China and Mongolia Ibis 2009 151 568 576 10.1111/j.1474-919X.2009.00932.x

[B33] Senne DA Suarez DL Stallnecht DE Pedersen JC Panigrahy B Schudel A, Lombard M Ecology and epidemiology of avian influenza in North and South America OIE/FAO International Scientific Conference on Avian Influenza 2006 Basel: Karger 37 44 16447492

[B34] Verdugo C Cardona CJ Carpenter TE Simulation of an early warning system using sentinel birds to detect a change of a low pathogenic avian influenza virus (LPAIV) to high pathogenic avian influenza virus (HPAIV) Prev Vet Med 2009 88 109 119 10.1016/j.prevetmed.2008.08.007 18977544

[B35] Gilbert M Xiao X Domenech J Lubroth J Martin V Slingenbergh J Anatidae migration in the western Palearctic and spread of highly pathogenic avian influenza H5N1 virus Emerging Infectious Diseases 2006 12 1650 1656 17283613 10.3201/eid1211.060223 PMC3372333

[B36] Krauss S Walker D Pryor SP Niles L Chenghong L Hinshaw VS Webster RG Influenza A viruses of migrating wild aquatic birds in North America Vector-borne and Zoonotic Diseases 2004 4 177 189 10.1089/vbz.2004.4.177 15631061

[B37] Hanson BA Luttrell MP Goekjian VH Niles L Swayne DE Senne DA Stallknecht DE Is the occurrence of avian influenza virus in Charadriiformes species and location dependent? Journal of Wildlife Diseases 2008 44 351 361 18436667 10.7589/0090-3558-44.2.351

[B38] Gilbert M Xiao X Pfeiffer DU Epprecht M Boles S Czarnecki C Chaitaweesub P Kalpravidh W Minh PQ Otte MJ Mapping H5N1 highly pathogenic avian influenza risk in Southeast Asia Proceedings of the National Academy of Sciences 2008 105 4769 4774 10.1073/pnas.0710581105 PMC2290786 18362346

[B39] Pearce JM Ramey AM Flint PL Koehler AV Fleskes JP Franson JC Hall JS Derksen DV Ip HS Avian influenza at both ends of a migratory flyway: characterizing viral genomic diversity to optimize surveillance plans for North America Evolutionary Applications 2009 2 457 468 10.1111/j.1752-4571.2009.00071.x PMC3352445 25567891

[B40] Dusek RJ Bortner JB DeLiberto TJ Hoskins J Franson JC Bales BD Yparraguirre D Swafford SR Ip HS Surveillance for high pathogenicity avian influenza virus in wild birds in the Pacific Flyway of the United States, 2006-2007 Avian Diseases 2009 53 222 230 10.1637/8462-082908-Reg.1 19630228

[B41] Pearce JM Ramey AM Ip HS Gill RE Limited evidence of trans-hemispheric movement of avian influenza viruses among contemporary North American shorebird isolates Virus Res 2010 148 44 50 10.1016/j.virusres.2009.12.002 19995585

[B42] Peterson AT Bush SE Spackman E Swayne DE Ip HS Influenza A virus infections in land birds, People's Republic of China Emerging Infectious Diseases 2008 14 1644 1646 10.3201/eid1410.080169 18826836 PMC2609895

[B43] Kou Z Lei FM Yu J Fan ZJ Yin ZH Jia CX Xiong KJ Sun YH Zhang XW Wu XM New genotype of avian influenza H5N1 viruses isolated from tree sparrows in China Journal of Virology 2005 79 15460 15466 10.1128/JVI.79.24.15460-15466.2005 16306617 PMC1316012

[B44] Lubroth J Morzaria S Thiermann AB Swayne DE Global strategy for highly pathogenic avian influenza: progressive control and eradication, and postoutbreak recovery Avian Influenza 2008 Ames, Iowa: Blackwell 561 585 full_text

[B45] Williams RAJ Fasina FO Peterson AT Predictable ecology and geography of avian influenza (H5N1) transmission in Nigeria and West Africa Transactions of the Royal Society of Tropical Medicine and Hygiene 2008 102 471 479 10.1016/j.trstmh.2008.01.016 18343470

[B46] Ip HS Flint PL Franson JC Dusek RJ Derksen DV Gill JRE Ely CR Pearce JM Lanctot RB Matsuoka SM Prevalence of influenza A in viruses in wild migratory birds in Alaska: patterns of variation in detection at a crossroads of intercontinental flyways Virology Journal 2008 5 71 doi:10.1186/1743-1422X-1185-1171 10.1186/1743-422X-5-71 18533040 PMC2435106

[B47] Winker K McCracken KG Gibson DD Pruett CL Meier R Huettmann F Wege M Kulikova IV Zhuravlev YN Perdue ML Movements of birds and avian influenza from Asia into Alaska Emerging Infectious Diseases 2007 13 547 552 10.3201/eid1304.061072 17553268 PMC2725966

[B48] Peterson AT Benz BW Papeş M Highly pathogenic H5N1 avian influenza: entry pathways into North America via bird migration PLoS One 2008 2 e261 10.1371/journal.pone.0000261 PMC1803015 17330144

[B49] Stallknecht DE Brown JD Swayne DE Ecology of avian influenza in wild birds Avian Influenza 2008 Ames, Iowa: Blackwell 43 58 full_text

[B50] Saracco JF Desante DF Kaschube DR Assessing landbird monitoring programs and demographic causes of population trends Journal of Wildlife Management 2008 72 1665 1673 10.2193/2008-129

[B51] Saracco JF Desante DF Nott MP Kaschube DR Rich TD, Thompson CD, Demarest D, Arizmendi C Using the MAPS and MoSI programs to monitor landbirds and inform conservation Proceedings of the Fourth International Partners in Flight Conference: Tundra to Tropics 2009 University of Texas-Pan American Press 659 661

[B52] Kalthoff D Breithaupt A Helm B Teifke JP Beer M Migratory status is not related to the susceptibility to HPAIV H5N1 in an insectivorous passerine species PLoS One 2009 4 e6170 10.1371/journal.pone.0006170 PMC2703776 19584935

[B53] Broussard W Turner RE A century of changing land-use and water-quality relationships in the continental US Frontiers in Ecology and the Environment 2009 7 302 307 10.1890/080085

[B54] Ward MP Maftei DN Apostu CL Suru AR Association between outbreaks of highly pathogenic avian influenza subtype H5N1 and migratory waterfowl (Family *Anatidae*) populations Zoonoses and Public Health 2008 56 1 9 10.1111/j.1863-2378.2008.01150.x 18793277

[B55] Gauthier-Clerc M Lebarbenchon C Thomas F Recent expansion of highly pathogenic avian influenza H5N1: a critical review Ibis 2007 149 202 214 10.1111/j.1474-919X.2007.00699.x

[B56] Wint W Robinson T Gridded Livestock of the World 2007 Rome: Food and Agriculture Organization of the United Nations 20422554

[B57] Daly C Halbleib M Smith JI Gibson WP Doggett MK Taylor GH Curtis J Pasteris PP Physiographically sensitive mapping of climatological temperature and precipitation across the conterminous United States Int J Climatol 2008 28 2031 2064 10.1002/joc.1688

[B58] Giese BS Compo GP Slowey PD Carton JA Ray S Whitaker JS The 1918/19 El Niño Bulletin of the American Meteorological Society 2010 91 177 183 10.1175/2009BAMS2903.1

[B59] Berthold P Bird Migration. A General Survey 2001 Second Oxford, UK: Oxford University Press

[B60] Cecchi G Ilemobade A Le Brun Y Hogerwerf L Slingenbergh J Agro-ecological features of the introduction and spread of the highly pathogenic avian influenza (HPAI) H5N1 in northern Nigeria Geospatial Health 2008 3 7 16 19021104 10.4081/gh.2008.227

[B61] Breban R Drake JM Stallknecht DE Rohani P The role of enviromental transmission in recurrent avian influenza epidemics PLoS Computational Biology 2009 5 e1000346 10.1371/journal.pcbi.1000346 19360126 PMC2660440

[B62] Smith NV Saatchi SS Randerson JT Trends in high northern latitude soil freeze and thaw cycles from 1988 to 2002 Journal of Geophysical Research-Atmospheres 2004 109

[B63] Xiao X Gilbert M Slingenbergh J Lei F Boles S Remote sensing, ecological variables, and wild bird migration related to outbreaks of highly pathogenic H5N1 avian influenza Journal of Wildlife Diseases 2007 43 S40 S46 PMC2735754 28813587

[B64] Montgomery DC Peck EA Vining GG An Introduction to Linear Regression Analysis 2006 Fourth17280293

[B65] Kleinschmidt I Sharp BL Clarke GPY Curtis B Fraser C Use of generalized linear mixed models in the spatial analysis of small-area malaria incidence rates in KwaZulu Natal, South Africa American Journal of Epidemiology 2001 153 1213 1221 10.1093/aje/153.12.1213 11415957

[B66] Cressie NAC Statistics for Spatial Data. Revised Edition 1993 New York: Wiley-Interscience

[B67] Schabenberger O Gotway CA Statistical Methods for Spatial Data Analysis 2005 Boca Raton: Chapman & Hall/CRC

[B68] Montgomery DC Peck EA Vining GG An Introduction to Linear Regression Analysis 2006 Fourth Hoboken, New Jersey: John Wiley & Sons

[B69] De Santis A Chuvieco E Burn severity estimation from remotely sensed data: performance of simulation versus empirical models Remote Sensing of Environment 2007 108 422 435 10.1016/j.rse.2006.11.022

[B70] Latorre-Margalef N Gunnarsson G Munster VJ Fouchier RAM Osterhaus ADME Elmberg J Olsen B Wallensten A Haemig PD Fransson T Effects of influenza A virus infection on migrating mallard ducks Proceedings of the Royal Society B 2009 276 1029 1036 10.1098/rspb.2008.1501 19129127 PMC2679067

[B71] Fouchier RAM Munster VJ Epidemiology of low pathogenic avian influenza viruses in wild birds Revue Scientifique et Technique (International Office of Epizootics) 2009 28 49 58 19618618 10.20506/rst.28.1.1863

[B72] Olsen B Munster VJ Wallensten A Waldenström J Osterhaus ADME Fouchier RAM Global patterns of influenza A virus in wild birds Science 2006 312 384 388 10.1126/science.1122438 16627734

[B73] Ito T Okazaki K Kawaoka Y Takada A Webster RG Kida H Perpetuation of influenza A viruses in Alaskan waterfowl reservoirs Arch Virol 1995 140 1163 1172 10.1007/BF01322743 7646350

[B74] Sims LD Brown IH Swayne DE Multicontinental epidemic of H5N1 HPAI virus (1996-2007) Avian Influenza 2008 Ames, Iowa: Blackwell 251 286 full_text

[B75] Komar N Olsen B Avian influenza virus (H5N1) mortality surveillance Emerging Infectious Diseases 2008 14 1176 1178 10.3201/eid1407.080161 18598659 PMC2600356

[B76] Halvorson DA Control of low pathogenicity avian influenza Avian Influenza 2008 Ames, Iowa: Blackwell 513 536 full_text

[B77] Koehler AV Pearce JM Flint PL Franson JC Ip HS Genetic evidence of intercontinental movement of avian influenza in a migratory bird: the northern pintail (*Anas acuta*) Molecular Ecology 2008 17 4754 4762 10.1111/j.1365-294X.2008.03953.x 19140989

[B78] Smith GJD Bahl J Vijaykrishna D Zhang JX Poon LLM Chen HL Webster RG Peiris JSM Guan Y Dating the emergence of pandemic influenza viruses Proc Natl Acad Sci USA 2009 106 11709 11712 10.1073/pnas.0904991106 19597152 PMC2709671

[B79] Cardona CJ Swayne DE Farm and regional biosecurity practices Avian Influenza 2008 Ames, Iowa: Blackwell 353 367 full_text

[B80] Dorian A Rottman SJ Shoaf K Tharian B The novel influenza A H1N1 epidemic of Spring 2009: national after action workshop on a federal public health emergency: 21-22 September 2009 Torrance, California Prehospital and Disaster Medicine 2009 http://pdm.medicine.wisc.edu/H1N1.pdf Web Exclusive Report, November 2009

[B81] Rogers DJ Randolph SE The global spread of malaria in a future, warmer world Science 2000 289 1763 1766 10.1126/science.289.5478.391b 10976072

[B82] Hanson BA Luttrell MP Goekjian VH Niles L Swayne DE Senne DA Stallknecht DE Is the occurrence of avian influenza virus dependent in Charadriiformes species and location dependent? Journal of Wildlife Diseases 2008 44 351 361 18436667 10.7589/0090-3558-44.2.351

[B83] Moffett A Shackelford N Sarkar S Malaria in Africa: vector species' niche models and relative risk maps PLoS One 2007 2 e824 10.1371/journal.pone.0000824 17786196 PMC1950570

[B84] Woodruff C Economic slowdown widespread among states in 2008. Advance 2008 and Revised 2005-2007 GDP-by-State Statistics. BEA09-22 2009 Washington, DC: US Department of Commerce, Bureau of Economic Analysis http://www.bea.gov/newsreleases/regional/gdp_state/2009/pdf/gsp0609.pdf

