# Supplementary material for: Mapping the risk of avian influenza in wild birds in the US
Source: BMC Infect Dis. 2010 Jun 23;10:187. doi: 10.1186/1471-2334-10-187 (PMC2912310; doi:10.1186/1471-2334-10-187)
Supplement: Additional file 1 — Influenza samples from wild birds used in the study. This file provides a detailed description of the geographical study region and lists the online databases from which we obtained samples in addition to the samples tested at the UCLA Center for Tropical Research. [file 1471-2334-10-187-S1.PDF]

Mapping the Risk of Avian Influenza in Wild Birds in the US

Additional File 1 – Influenza Samples from Wild Birds Used in this Study

Study region

The contiguous US was divided into 3109 counties or county equivalents (boroughs, census areas, and parishes) using the US contiguous Albers Equal Area Conic Projection. We selected this projection because it allows accurate areal calculations. The average area of the counties was 2505.6 km^2 +/- 3403.87 (mean +/- standard deviation).

Analysis of existing influenza samples from wild birds

To increase the geographic region sampled by the AIV case data, we obtained additional records from two online databases: the NIAID BioHealthBase BRC online through the web site at http://www.biohealthbase.org and the USDA (44) (Table 1).

Table 1 - Sources of AIV data

**Source**	**Number of samples**
BioHealthBase	7745
Monitoring Avian Productivity and Survival (MAPS) network at the Institute for Bird Populations (IBP) in collaboration with the Center for Tropical Research (CTR), University of California, Los Angeles	5233
USDA	68
**Total**	**13,046**
