# Supplementary material for: Mapping the risk of avian influenza in wild birds in the US
Source: BMC Infect Dis. 2010 Jun 23;10:187. doi: 10.1186/1471-2334-10-187 (PMC2912310; doi:10.1186/1471-2334-10-187)
Supplement: Additional file 2 — Description of the samples by species. The table in this file reports the prevalence of flu in the 225 avian species analyzed in this study, which represent 11 orders of birds. [file 1471-2334-10-187-S2.PDF]

# Mapping the Risk of Avian Influenza in Wild Birds in the US

## Additional File 2 – Description of the Samples by Species

**Table 1 - Description of the samples by species. Prevalence is defined as the % of samples collected from the species that tested positive for AIV. Among the 225 species analyzed here, which represent 11 orders of birds, Anseriformes, Charadriiformes, and Passeriformes had the highest average prevalence of influenza. In particular, species in the order Passeriformes exhibited higher prevalence than species from eight other avian orders. The influenza data set analyzed in the main text comprises 13,046 samples from 225 species. For 5.62 % of the samples, the day of the year when the first or last sample was collected was not available, although all samples were collected between December 2005 and 2008. This table summarizes the 12,313 samples for which the first and last collection date was available. Taxonomy is based on the list of North American bird species maintained by the Cornell Bird Lab available from http://www.allaboutbirds.org.**

| **Scientific Name** | **Common Name** | **Number of Samples** | **Prevalence (%)** | **First Collection Date** | **Last Collection Date** |
|---|---|---|---|---|---|
| Anseriformes (*n*=30) | | | | | |
| *Aix sponsa* | Wood Duck | 374 | 0 | 2007-Jul-19 | 2008-Jan-23 |
| *Anas acuta* | Northern Pintail | 467 | 3 | 2006-Sep-21 | 2008-Nov-04 |
| *Anas Americana* | American Wigeon | 361 | 2.77 | 2007-Aug-01 | 2008-Nov-06 |
| *Anas carolinensis* | Green-winged Teal | 163 | 7.36 | 2006-Sep-24 | 2007-Nov-10 |
| *Anas clypeata* | Northern Shoveler | 560 | 4.11 | 2006-Nov-04 | 2008-Nov-12 |
| *Anas crecca* | Common Teal | 468 | 0 | 2007-Sep-19 | 2008-Jan-27 |
| *Anas cyanoptera* | Cinnamon Teal | 60 | 3.33 | 2007-Sep-16 | 2008-Jan-23 |
| *Anas discors* | Blue-winged Teal | 1045 | 5.45 | 2007-Jul-16 | 2008-Sep-15 |
| *Anas Penelope* | Eurasian Wigeon | 2 | 0 | 2008-Jan-23 | 2008-Jan-26 |
| *Anas platyrhynchos* | Mallard | 2255 | 5.9 | 2006-Aug-02 | 2008-Dec-11 |
| *Anas rubripes* | American Black Duck | 10 | 50 | 2006-Dec-19 | 2008-Oct-19 |
| *Anas strepera* | Gadwall | 402 | 0.498 | 2007-Aug-01 | 2008-Jan-26 |
| *Anser albifrons* | Greater White-fronted Goose | 82 | 1.22 | 2007-Oct-21 | 2008-Jan-27 |
| *Aythya affinis* | Lesser Scaup | 59 | 0 | 2007-Oct-26 | 2008-Jan-23 |

| Scientific Name | Common Name | Number of Samples | Prevalence (%) | First Collection Date | Last Collection Date |
|---|---|---|---|---|---|
| *Aythya Americana* | Redhead | 3 | 33.3 | 2007-Sep-29 | 2007-Sep-30 |
| *Aythya collaris* | Ring-necked Duck | 170 | 4.12 | 2007-Aug-01 | 2008-Jan-23 |
| *Aythya marila* | Greater Scaup | 3 | 0 | 2007-Oct-26 | 2007-Nov-04 |
| *Aythya valisineria* | Canvasback | 62 | 0 | 2007-Aug-01 | 2008-Jan-26 |
| *Branta Canadensis* | Canada Goose | 17 | 5.88 | 2007-Oct-20 | 2008-Dec-29 |
| *Branta hutchinsii* | Cackling Goose | 11 | 0 | 2007-Oct-27 | 2008-Jan-16 |
| *Bucephala albeola* | Bufflehead | 45 | 2.22 | 2007-Aug-03 | 2008-Jan-23 |
| *Bucephala clangula* | Common Goldeneye | 29 | 0 | 2007-Jul-12 | 2008-Jan-23 |
| *Chen caerulescens* | Snow Goose | 56 | 1.79 | 2007-Oct-28 | 2008-Jan-23 |
| *Chen rossii* | Ross's Goose | 14 | 0 | 2007-Nov-03 | 2008-Jan-26 |
| *Cygnus buccinators* | Trumpeter Swan | 19 | 0 | 2007-Aug-08 | 2008-Feb-02 |
| *Cygnus columbianus* | Tundra Swan | 11 | 0 | 2007-Nov-16 | 2008-Jan-02 |
| *Cygnus olor* | Mute Swan | 1 | 100 | 2006-Aug-08 | 2006-Aug-08 |
| *Lophodytes cucullatus* | Hooded Merganser | 3 | 0 | 2007-Aug-03 | 2007-Aug-06 |
| *Mergus merganser* | Common Merganser | 18 | 0 | 2007-Jul-13 | 2008-Jan-05 |
| *Oxyura jamaicensis* | Ruddy Duck | 30 | 0 | 2007-Oct-24 | 2008-Jan-23 |

| Scientific Name | Common Name | Number of Samples | Prevalence (%) | First Collection Date | Last Collection Date |
|---|---|---|---|---|---|
| **Mean (Anseriformes)** | | **226.67** | **7.698** | | |
| **Median (Anseriformes)** | | **50.5** | **0.859** | | |
| Charadriiformes (*n*=14) | | | | | |
| *Arenaria interpres* | Ruddy Turnstone | 43 | 16.3 | 2007-May-10 | 2007-May-22 |
| *Calidris alba* | Sanderling | 53 | 0 | 2007-May-22 | 2007-May-23 |
| *Calidris mauri* | Western Sandpiper | 22 | 0 | 2006-Jul-22 | 2006-Aug-19 |
| *Calidris minutilla* | Least Sandpiper | 1 | 0 | 2007-Nov-02 | 2007-Nov-02 |
| *Calidris pusilla* | Semipalmated Sandpiper | 83 | 0 | 2007-May-22 | 2008-May-23 |
| *Charadrius semipalmatus* | Semipalmated Plover | 1 | 0 | 2006-Aug-19 | 2006-Aug-19 |
| *Himantopus mexicanus* | Black-necked Stilt | 8 | 0 | 2006-Aug-19 | 2006-Aug-19 |
| *Larus argentatus* | Mongolian Gull | 7 | 0 | 2007-May-22 | 2007-May-22 |
| *Larus atricilla* | Laughing Gull | 23 | 0 | 2007-May-22 | 2007-May-24 |
| *Larus belcheri* | Belcher’s Gull | 1 | 0 | 2006-Aug-19 | 2006-Aug-19 |
| *Larus delawarensis* | Ring-Billed Gull | 5 | 0 | 2007-Jul-12 | 2007-Jul-12 |
| *Phalaropus lobatus* | Red-necked | 3 | 0 | 2006-Jul-22 | 2006-Aug-18 |

| Scientific Name | Common Name | Number of Samples | Prevalence (%) | First Collection Date | Last Collection Date |
|---|---|---|---|---|---|
| | Phalarope | | | | |
| *Phalaropus tricolor* | Wilson's Phalarope | 8 | 0 | 2006-Jul-22 | 2006-Aug-18 |
| *Recurvirostra americana* | American Avocet | 5 | 0 | 2006-Aug-19 | 2006-Aug-19 |
| **Mean (Charadriiformes)** | | **18.786** | **1.164** | | |
| **Median (Charadriiformes)** | | **7.5** | **0** | | |
| Ciconiiformes (*n*=3) | | | | | |
| *Cathartes aura* | Turkey Vulture | 28 | 0 | 2007-Sep-26 | 2008-Mar-04 |
| *Coragyps atratus* | Black Vulture | 6 | 0 | 2007-Oct-11 | 2008-Feb-05 |
| *Egretta gularis* | Western Reef-Heron | 8 | 0 | 2007-Nov-03 | 2007-Dec-02 |
| **Mean (Ciconiiformes)** | | **14** | **0** | | |
| **Median (Ciconiiformes)** | | **8** | **0** | | |
| Columbiformes (*n*=2) | | | | | |
| *Columbina passerina* | Common Ground-Dove | 2 | 0 | 2005-Dec-07 | 2005-Dec-24 |
| *Zenaida macroura* | Mourning Dove | 4 | 0 | 2005-Dec-14 | 2008-Jun-30 |
| **Mean (Columbiformes)** | | **3** | **0** | | |

| Scientific Name | Common Name | Number of Samples | Prevalence (%) | First Collection Date | Last Collection Date |
|---|---|---|---|---|---|
| **Median (Columbiformes)** | | **3** | **0** | | |
| Cuculiformes | | | | | |
| *Coccyzus americanus* | Yellow-billed Cuckoo | 1 | 0 | 2008-Jul-01 | 2008-Jul-01 |
| Falconiformes (*n*=4) | | | | | |
| *Accipiter cooperii* | Cooper’s Hawk | 62 | 0 | 2007-Oct-08 | 2008-Mar-08 |
| *Accipitridae striatus* | Sharp-shinned Hawk | 1 | 0 | 2008-May-13 | 2008-May-13 |
| *Falco peregrines* | Peregrine Falcon | 126 | 0 | 2007-Sep-30 | 2007-Dec-18 |
| *Haliaeetus leucocephalus* | Bald Eagle | 81 | 1.23 | 2007-Sep-27 | 2008-Mar-16 |
| **Mean (Falconiformes)** | | **67.5** | **0.3075** | | |
| **Median (Falconiformes)** | | **71.5** | **0** | | |
| Gruiformes | | | | | |
| *Rallus elegans* | King Rail | 1 | 0 | 2007-Sep-18 | 2007-Sep-18 |
| Passeriformes (*n*=152) | | | | | |
| *Agelaius phoeniceus* | Red-winged Blackbird | 21 | 0 | 2005-Dec-07 | 2008-Jul-18 |
| *Aimophila ruficeps* | Rufous-crowned | 10 | 0 | 2008-Jun-19 | 2008-Jul-19 |

| Scientific Name | Common Name | Number of Samples | Prevalence (%) | First Collection Date | Last Collection Date |
|---|---|---|---|---|---|
| | Sparrow | | | | |
| *Amphispiza bilineata* | Black-throated Sparrow | 2 | 0 | 2008-Jun-19 | 2008-Sep-06 |
| *Aphelocoma californica* | Western Scrub-Jay | 1 | 0 | 2005-Dec-07 | 2005-Dec-07 |
| *Baeolophus atricristatus* | Black-crested Titmouse | 2 | 0 | 2008-Jul-01 | 2008-Jul-03 |
| *Baeolophus bicolor* | Tufted Titmouse | 83 | 0 | 2005-Dec-02 | 2008-Oct-13 |
| *Baeolophus inornatus* | Oak Titmouse | 1 | 0 | 2005-Dec-03 | 2005-Dec-03 |
| *Bombycilla cedrorum* | Cedar Waxwing | 33 | 3.03 | 2005-Dec-04 | 2008-Sep-08 |
| *Cardinalis cardinalis* | Northern Cardinal | 147 | 0 | 2005-Dec-03 | 2008-Sep-21 |
| *Cardinalis sinuatus* | Pyrrhuloxia | 1 | 0 | 2008-Jun-19 | 2008-Jun-19 |
| *Carduelis pinus* | Pine Siskin | 10 | 0 | 2008-Jun-14 | 2008-Jul-03 |
| *Carduelis psaltria* | Lesser Goldfinch | 1 | 0 | 2008-Aug-01 | 2008-Aug-01 |
| *Carduelis tristis* | American Goldfinch | 36 | 2.78 | 2005-Dec-11 | 2008-Sep-08 |
| *Carpodacus cassinii* | Cassin’s Finch | 12 | 8.33 | 2005-Dec-03 | 2008-Jun-14 |
| *Carpodacus mexicanus* | House Finch | 14 | 0 | 2005-Dec-04 | 2008-Jul-28 |
| *Carpodacus purpureus* | Purple Finch | 69 | 0 | 2005-Dec-01 | 2008-Sep-04 |

| Scientific Name | Common Name | Number of Samples | Prevalence (%) | First Collection Date | Last Collection Date |
|---|---|---|---|---|---|
| *Catharus bicknelli* | Bicknell's Thrush | 2 | 0 | 2008-Jun-05 | 2008-Sep-23 |
| *Catharus fuscescens* | Veery | 19 | 5.26 | 2006-Jul-04 | 2008-Sep-14 |
| *Catharus guttatus* | Hermit Thrush | 103 | 0.97 | 2005-Dec-01 | 2008-Oct-19 |
| *Catharus minimus* | Gray-cheeked Thrush | 2 | 0 | 2008-May-27 | 2008-May-28 |
| *Catharus ustulatus* | Swainson's Thrush | 265 | 3.77 | 2005-Dec-01 | 2008-Oct-11 |
| *Catherpes mexicanus* | Canyon Wren | 3 | 0 | 2008-Jun-19 | 2008-Jul-19 |
| *Certhia americana* | Brown Creeper | 3 | 0 | 2008-Jun-16 | 2008-Jul-30 |
| *Chamaea fasciata* | Wrentit | 14 | 0 | 2005-Dec-03 | 2008-Jul-27 |
| *Chondestes grammacus* | Lark Sparrow | 2 | 0 | 2008-Jul-03 | 2008-Jul-05 |
| *Coccothraustes vespertinus* | Evening Grosbeak | 7 | 0 | 2005-Dec-01 | 2005-Dec-30 |
| *Contopus cooperi* | Olive-sided Flycatcher | 2 | 0 | 2005-Dec-20 | 2008-Jul-25 |
| *Contopus sordidulus* | Western Wood-Pewee | 22 | 4.55 | 2005-Dec-12 | 2008-Jul-22 |
| *Contopus virens* | Eastern Wood-Pewee | 15 | 0 | 2007-Dec-01 | 2008-Sep-18 |

| Scientific Name | Common Name | Number of Samples | Prevalence (%) | First Collection Date | Last Collection Date |
|---|---|---|---|---|---|
| *Cyanocitta cristata* | Blue Jay | 15 | 0 | 2005-Dec-03 | 2008-Sep-14 |
| *Cyanocitta stelleri* | Steller’s Jay | 8 | 0 | 2005-Dec-02 | 2008-Jul-27 |
| *Dendroica caerulescens* | Black-throated Blue Warbler | 22 | 0 | 2007-Dec-31 | 2008-Oct-12 |
| *Dendroica cerulean* | Cerulean Warbler | 1 | 0 | 2008-Jun-21 | 2008-Jun-21 |
| *Dendroica coronate* | Yellow-rumped Warbler | 50 | 4 | 2005-Dec-02 | 2008-Oct-12 |
| *Dendroica discolor* | Prairie Warbler | 23 | 0 | 2005-Dec-04 | 2008-Jul-02 |
| *Dendroica dominica* | Yellow-throated Warbler | 1 | 0 | 2008-Jun-21 | 2008-Jun-21 |
| *Dendroica magnolia* | Magnolia Warbler | 28 | 0 | 2007-Dec-07 | 2008-Oct-08 |
| *Dendroica occidentalis* | Hermit Warbler | 4 | 0 | 2005-Dec-02 | 2008-Jun-30 |
| *Dendroica pensylvanica* | Chestnut-sided Warbler | 5 | 0 | 2007-Dec-10 | 2008-Sep-24 |
| *Dendroica petechia* | Yellow Warbler | 82 | 0 | 2005-Dec-03 | 2008-Sep-06 |
| *Dendroica pharetra* | Arrowhead Warbler | 1 | 0 | 2005-Dec-01 | 2005-Dec-01 |
| *Dendroica striata* | Blackpoll Warbler | 2 | 0 | 2008-May-27 | 2008-May-28 |

| Scientific Name | Common Name | Number of Samples | Prevalence (%) | First Collection Date | Last Collection Date |
|---|---|---|---|---|---|
| *Dendroica townsendi* | Townsend's Warbler | 9 | 0 | 2005-Dec-02 | 2008-Jul-25 |
| *Dendroica virens* | Black-throated Green Warbler | 1 | 0 | 2008-Sep-22 | 2008-Sep-22 |
| *Dumetella carolinensis* | Gray Catbird | 251 | 0 | 2005-Dec-05 | 2008-Sep-22 |
| *Empidonax alnorum* | Alder Flycatcher | 25 | 4 | 2007-Dec-31 | 2008-Sep-21 |
| *Empidonax difficilis* | Pacific-slope Flycatcher | 16 | 6.25 | 2005-Dec-02 | 2008-Jul-25 |
| *Empidonax flaviventris* | Yellow-bellied Flycatcher | 8 | 0 | 2007-May-19 | 2008-Sep-19 |
| *Empidonax hammondii* | Hammond's Flycatcher | 3 | 0 | 2005-Dec-06 | 2008-Jul-12 |
| *Empidonax minimus* | Least Flycatcher | 1 | 0 | 2008-May-31 | 2008-May-31 |
| *Empidonax oberholseri* | Dusky Flycatcher | 24 | 4.17 | 2005-Dec-03 | 2008-Sep-24 |
| *Empidonax traillii* | Willow Flycatcher | 25 | 0 | 2005-Dec-08 | 2008-Jul-31 |
| *Empidonax virescens* | Acadian Flycatcher | 46 | 0 | 2007-Dec-01 | 2008-Jul-30 |
| *Empidonax wrightii* | Gray Flycatcher | 3 | 0 | 2005-Dec-05 | 2008-Jun-03 |
| *Geothlypis trichas* | Common Yellowthroat | 160 | 0 | 2007-Dec-01 | 2008-Oct-03 |

| Scientific Name | Common Name | Number of Samples | Prevalence (%) | First Collection Date | Last Collection Date |
|---|---|---|---|---|---|
| *Helmitheros vermivorus* | Worm-eating Warbler | 15 | 0 | 2007-Dec-24 | 2008-Jul-19 |
| *Hirundo rustica* | Barn Swallow | 4 | 0 | 2008-Jun-22 | 2008-Aug-03 |
| *Hylocichla mustelina* | Wood Thrush | 183 | 0 | 2005-Dec-18 | 2008-Sep-14 |
| *Icteria virens* | Yellow-breasted Chat | 92 | 1.09 | 2005-Dec-11 | 2008-Sep-12 |
| *Icterus bullockii* | Bullock's Oriole | 28 | 0 | 2005-Dec-17 | 2008-Aug-26 |
| *Icterus cucullatus* | Hooded Oriole | 1 | 0 | 2005-Dec-13 | 2005-Dec-13 |
| *Icterus galbula* | Baltimore Oriole | 12 | 0 | 2007-Dec-31 | 2008-Jun-24 |
| *Icterus spurious* | Orchard Oriole | 2 | 0 | 2008-May-25 | 2008-May-25 |
| *Ixoreus naevius* | Varied Thrush | 14 | 0 | 2005-Dec-03 | 2008-Aug-03 |
| *Junco hyemalis* | Dark-eyed Junco | 125 | 3.2 | 2005-Dec-01 | 2008-Oct-13 |
| *Melospiza georgiana* | Swamp Sparrow | 22 | 0 | 2008-May-07 | 2008-Oct-19 |
| *Melospiza lincolnii* | Lincoln's Sparrow | 31 | 0 | 2005-Dec-04 | 2008-Oct-12 |
| *Melospiza melodia* | Song Sparrow | 263 | 0.76 | 2005-Dec-01 | 2008-Oct-19 |
| *Mimus polyglottos* | Northern Mockingbird | 4 | 0 | 2005-Dec-03 | 2007-Dec-04 |

| Scientific Name | Common Name | Number of Samples | Prevalence (%) | First Collection Date | Last Collection Date |
|---|---|---|---|---|---|
| *Mniotilta varia* | Black-and-white Warbler | 22 | 0 | 2007-Sep-09 | 2008-Sep-14 |
| *Molothrus ater* | Brown-headed Cowbird | 26 | 0 | 2005-Dec-07 | 2008-Jul-01 |
| *Myadestes townsendi* | Townsend's Solitaire | 3 | 0 | 2005-Dec-03 | 2005-Dec-23 |
| *Myiarchus cinerascens* | Ash-throated Flycatcher | 5 | 0 | 2005-Dec-01 | 2008-Jun-29 |
| *Myiarchus crinitus* | Great Crested Flycatcher | 8 | 0 | 2005-Dec-17 | 2008-Jul-14 |
| *Oporornis agilis* | Connecticut Warbler | 2 | 0 | 2008-Sep-14 | 2008-Oct-12 |
| *Oporornis formosus* | Kentucky Warbler | 120 | 0 | 2007-Dec-01 | 2008-Jul-30 |
| *Oporornis philadelphia* | Mourning Warbler | 6 | 0 | 2007-Dec-15 | 2008-Jun-05 |
| *Oporornis tolmiei* | MacGillivray's Warbler | 80 | 2.5 | 2005-Dec-01 | 2008-Sep-07 |
| *Parula americana* | Northern Parula | 4 | 0 | 2007-Dec-25 | 2008-Jun-21 |
| *Passerella iliaca* | Fox Sparrow | 10 | 10 | 2005-Dec-01 | 2008-Sep-12 |
| *Passerina versicolor* | Varied Bunting | 2 | 0 | 2008-Jun-09 | 2008-Jun-19 |

| Scientific Name | Common Name | Number of Samples | Prevalence (%) | First Collection Date | Last Collection Date |
|---|---|---|---|---|---|
| *Passerina amoena* | Lazuli Bunting | 9 | 0 | 2005-Dec-06 | 2008-Aug-01 |
| *Passerina caerulea* | Blue Grosbeak | 7 | 0 | 2008-Jun-03 | 2008-Aug-31 |
| *Passerina ciris* | Painted Bunting | 25 | 0 | 2005-Dec-04 | 2008-Jul-19 |
| *Passerina cyanea* | Indigo Bunting | 116 | 0 | 2005-Dec-29 | 2008-Aug-05 |
| *Perisoreus canadensis* | Gray Jay | 2 | 0 | 2005-Dec-05 | 2005-Dec-17 |
| *Pheucticus ludovicianus* | Rose-breasted Grosbeak | 9 | 0 | 2007-Dec-06 | 2008-Jun-28 |
| *Pheucticus melanocephalus* | Black-headed Grosbeak | 78 | 1.28 | 2005-Dec-01 | 2008-Aug-03 |
| *Phylloscopus sibilatrix* | Wood Warbler | 1 | 0 | 2008-Jun-10 | 2008-Jun-10 |
| *Pinicola enucleator* | Pine Grosbeak | 1 | 0 | 2008-Jun-22 | 2008-Jun-22 |
| *Pipilo chlorurus* | Green-tailed Towhee | 6 | 0 | 2008-Sep-04 | 2008-Sep-17 |
| *Pipilo erythrophthalmus* | Eastern Towhee | 29 | 0 | 2005-Dec-01 | 2008-Aug-03 |
| *Pipilo maculates* | Spotted Towhee | 41 | 0 | 2005-Dec-01 | 2008-Aug-01 |
| *Piranga ludoviciana* | Western Tanager | 55 | 9.09 | 2005-Dec-01 | 2008-Sep-09 |
| *Piranga olivacea* | Scarlet Tanager | 13 | 0 | 2007-Dec-27 | 2008-Sep-08 |

| Scientific Name | Common Name | Number of Samples | Prevalence (%) | First Collection Date | Last Collection Date |
|---|---|---|---|---|---|
| *Piranga rubra* | Summer Tanager | 15 | 0 | 2005-Dec-29 | 2008-Jul-03 |
| *Pitangus sulphuratus* | Great Kiskadee | 1 | 0 | 2008-Jul-12 | 2008-Jul-12 |
| *Poecile atricapillus* | Black-capped Chickadee | 50 | 0 | 2005-Dec-11 | 2008-Oct-11 |
| *Poecile carolinensis* | Carolina Chickadee | 18 | 0 | 2007-Dec-25 | 2008-Oct-12 |
| *Poecile gambeli* | Mountain Chickadee | 14 | 0 | 2005-Dec-06 | 2008-Jul-22 |
| *Poecile rufescens* | Chestnut-backed Chickadee | 5 | 0 | 2005-Dec-03 | 2008-Jul-24 |
| *Polioptila caerulea* | Blue-gray Gnatcatcher | 1 | 0 | 2008-Jun-25 | 2008-Jun-25 |
| *Protonotaria citrea* | Prothonotary Warbler | 3 | 0 | 2008-Jun-06 | 2008-Aug-03 |
| *Quiscalus quiscula* | Common Grackle | 1 | 0 | 2008-Jul-10 | 2008-Jul-10 |
| *Regulus calendula* | Ruby-crowned Kinglet | 2 | 0 | 2006-Apr-05 | 2007-Dec-12 |
| *Regulus satrapa* | Golden-crowned Kinglet | 2 | 50 | 2005-Dec-01 | 2006-Aug-02 |
| *Sayornis nigricans* | Black Phoebe | 1 | 0 | 2005-Dec-09 | 2005-Dec-09 |

| Scientific Name | Common Name | Number of Samples | Prevalence (%) | First Collection Date | Last Collection Date |
|---|---|---|---|---|---|
| *Sayornis phoebe* | Eastern Phoebe | 16 | 0 | 2007-Dec-20 | 2008-Oct-04 |
| *Seiurus aurocapillus* | Ovenbird | 88 | 0 | 2007-Dec-02 | 2008-Sep-25 |
| *Seiurus motacilla* | Louisiana Waterthrush | 38 | 0 | 2008-May-23 | 2008-Jul-10 |
| *Seiurus noveboracensis* | Northern Waterthrush | 11 | 9.09 | 2006-Aug-05 | 2008-May-31 |
| *Setophaga ruticilla* | American Redstart | 60 | 0 | 2005-Dec-04 | 2008-Sep-14 |
| *Sialia mexicana* | Western Bluebird | 1 | 0 | 2005-Dec-23 | 2005-Dec-23 |
| *Sialia sialis* | Eastern Bluebird | 4 | 0 | 2005-Dec-30 | 2008-Jul-14 |
| *Sitta canadensis* | Red-breasted Nuthatch | 14 | 0 | 2005-Dec-03 | 2008-Sep-20 |
| *Sitta carolinensis* | White-breasted Nuthatch | 14 | 0 | 2005-Dec-12 | 2008-Oct-12 |
| *Spinus pinus* | Pine Siskin | 4 | 0 | 2005-Dec-03 | 2005-Dec-31 |
| *Spinus psaltria* | Lesser Goldfinch | 6 | 0 | 2005-Dec-01 | 2005-Dec-12 |
| *Spizella breweri* | Brewer’s Sparrow | 4 | 0 | 2008-Aug-20 | 2008-Sep-13 |
| *Spizella pallid* | Clay-colored Sparrow | 1 | 0 | 2008-Aug-24 | 2008-Aug-24 |

| **Scientific Name** | **Common Name** | **Number of Samples** | **Prevalence (%)** | **First Collection Date** | **Last Collection Date** |
|---|---|---|---|---|---|
| *Spizella passerine* | Chipping Sparrow | 44 | 0 | 2005-Dec-01 | 2008-Oct-01 |
| *Spizella pusilla* | Field Sparrow | 27 | 0 | 2007-Dec-02 | 2008-Jul-21 |
| *Stelgidopteryx serripennis* | Northern Rough-winged Swallow | 2 | 0 | 2008-May-24 | 2008-May-24 |
| *Tachycineta bicolor* | Tree Swallow | 1 | 0 | 2008-Jun-10 | 2008-Jun-10 |
| *Teretistris fornsi* | Oriente Warbler | 1 | 0 | 2007-Dec-21 | 2007-Dec-21 |
| *Thryomanes bewickii* | Bewick's Wren | 16 | 6.25 | 2005-Dec-23 | 2008-Aug-24 |
| *Thryothorus ludovicianus* | Carolina Wren | 74 | 0 | 2005-Dec-02 | 2008-Aug-03 |
| *Toxostoma rufum* | Brown Thrasher | 27 | 0 | 2005-Dec-03 | 2008-Jul-27 |
| *Troglodytes aedon* | House Wren | 22 | 0 | 2005-Dec-04 | 2008-Sep-21 |
| *Troglodytes troglodytes* | Winter Wren | 1 | 0 | 2008-Jul-02 | 2008-Jul-02 |
| *Turdus migratorius* | American Robin | 133 | 3.76 | 2005-Dec-01 | 2008-Oct-08 |
| *Tyrannus tyrannus* | Eastern Kingbird | 1 | 0 | 2005-Dec-07 | 2005-Dec-07 |
| *Tyrannus verticalis* | Western Kingbird | 1 | 0 | 2005-Dec-28 | 2005-Dec-28 |
| *Vermivora virginiae* | Virginia's Warbler | 4 | 0 | 2008-Aug-20 | 2008-Sep-10 |
| *Vermivora celata* | Orange-crowned Warbler | 25 | 0 | 2005-Dec-30 | 2008-Sep-04 |

| Scientific Name | Common Name | Number of Samples | Prevalence (%) | First Collection Date | Last Collection Date |
| --- | --- | --- | --- | --- | --- |
| *Vermivora peregrina* | Tennessee Warbler | 3 | 0 | 2007-Dec-04 | 2007-Dec-30 |
| *Vermivora pinus* | Blue-winged Warbler | 34 | 0 | 2007-Dec-01 | 2008-Sep-11 |
| *Vermivora ruficapilla* | Nashville Warbler | 22 | 0 | 2005-Dec-03 | 2008-Oct-07 |
| *Vireo cassinii* | Cassin's Vireo | 5 | 0 | 2005-Dec-31 | 2008-Jul-22 |
| *Vireo flavifrons* | Yellow-throated Vireo | 2 | 0 | 2008-May-27 | 2008-May-27 |
| *Vireo gilvus* | Warbling Vireo | 48 | 0 | 2005-Dec-01 | 2008-Sep-10 |
| *Vireo griseus* | White-eyed Vireo | 72 | 0 | 2005-Dec-24 | 2008-Sep-14 |
| *Vireo huttoni* | Hutton's Vireo | 1 | 0 | 2008-May-06 | 2008-May-06 |
| *Vireo olivaceus* | Red-eyed Vireo | 77 | 0 | 2005-Dec-31 | 2008-Oct-03 |
| *Vireo philadelphicus* | Philadelphia Vireo | 2 | 0 | 2008-May-25 | 2008-Oct-12 |
| *Vireo solitaries* | Blue-headed Vireo | 1 | 0 | 2008-May-05 | 2008-May-05 |
| *Wilsonia canadensis* | Canada Warbler | 6 | 0 | 2007-Dec-31 | 2008-Aug-26 |
| *Wilsonia citrine* | Hooded Warbler | 17 | 0 | 2008-May-23 | 2008-Jul-22 |
| *Wilsonia pusilla* | Wilson's Warbler | 45 | 0 | 2005-Dec-29 | 2008-Oct-07 |
| *Zonotrichia albicollis* | White-throated | 49 | 0 | 2005-Dec-01 | 2008-Oct-19 |

| Scientific Name | Common Name | Number of Samples | Prevalence (%) | First Collection Date | Last Collection Date |
|---|---|---|---|---|---|
| | Sparrow | | | | |
| *Zonotrichia atricapilla* | Golden-crowned Sparrow | 1 | 0 | 2008-Sep-10 | 2008-Sep-10 |
| *Zonotrichia leucophrys* | White-crowned Sparrow | 11 | 0 | 2005-Dec-10 | 2008-Jul-12 |
| **Mean (Passerines)** | | **28.559** | **0.948** | | |
| **Median (Passerines)** | | **10.5** | **0** | | |
| Pelecaniformes (*n*=2) | | | | | |
| *Pelecanus erythrorhynchos* | American White Pelican | 29 | 0 | 2007-Jul-12 | 2007-Jul-12 |
| *Phalacrocorax auritus* | Double-crested Cormorant | 4 | 0 | 2007-Jul-12 | 2007-Jul-12 |
| **Mean (Pelecaniformes)** | | **16.5** | **0** | | |
| **Median (Pelecaniformes)** | | **16.5** | **0** | | |
| Piciformes (*n*=14) | | | | | |
| *Colaptes auratus* | Northern Flicker | 20 | 0 | 2005-Dec-04 | 2008-Oct-03 |
| *Dryocopus pileatus* | Pileated Woodpecker | 1 | 0 | 2008-Jul-14 | 2008-Jul-14 |

| Scientific Name | Common Name | Number of Samples | Prevalence (%) | First Collection Date | Last Collection Date |
|---|---|---|---|---|---|
| *Melanerpes carolinus* | Red-bellied Woodpecker | 4 | 0 | 2005-Dec-18 | 2008-Oct-13 |
| *Melanerpes erythrocephalus* | Red-headed Woodpecker | 1 | 0 | 2005-Dec-30 | 2005-Dec-30 |
| *Picoides arcticus* | Black-backed Woodpecker | 1 | 0 | 2008-Jun-12 | 2008-Jun-12 |
| *Picoides nuttallii* | Nuttall's Woodpecker | 1 | 0 | 2005-Dec-06 | 2005-Dec-06 |
| *Picoides pubescens* | Downy Woodpecker | 23 | 0 | 2005-Dec-15 | 2008-Jul-20 |
| *Picoides scalaris* | Ladder-backed Woodpecker | 2 | 0 | 2008-Jul-09 | 2008-Jul-09 |
| *Picoides villosus* | Hairy Woodpecker | 13 | 0 | 2005-Dec-01 | 2008-Jul-14 |
| *Sphyrapicus nuchalis* | Red-naped Sapsucker | 8 | 0 | 2005-Dec-02 | 2008-Jun-16 |
| *Sphyrapicus nuchalis* x *ruber* | Red-naped X Red-breasted Sapsucker Hybrid | 4 | 0 | 2007-Dec-14 | 2008-Jul-03 |
| *Sphyrapicus ruber* | Red-breasted Sapsucker | 22 | 0 | 2005-Dec-02 | 2008-Jul-25 |

| Scientific Name | Common Name | Number of Samples | Prevalence (%) | First Collection Date | Last Collection Date |
|---|---|---|---|---|---|
| *Sphyrapicus thyroideus* | Williamson's Sapsucker | 4 | 0 | 2005-Dec-05 | 2008-Jun-22 |
| *Sphyrapicus varius* | Yellow-bellied Sapsucker | 1 | 0 | 2008-Sep-18 | 2008-Sep-18 |
| **Mean (Piciformes)** | | **7.5** | **0** | | |
| **Median (Piciformes)** | | **4** | **0** | | |
| Strigiformes | | | | | |
| *Bubo virginianus* | Great Horned Owl | 69 | 0 | 2007-Oct-04 | 2008-Mar-11 |
| Environmental | None | 382 | 0 | 2007-May-22 | 2008-May-24 |
| **Mean (all orders)** | | **54.72** | **1.75** | | |
| **Median (all orders)** | | **11** | **0** | | |
