# Supplementary material for: Mapping the risk of avian influenza in wild birds in the US
Source: BMC Infect Dis. 2010 Jun 23;10:187. doi: 10.1186/1471-2334-10-187 (PMC2912310; doi:10.1186/1471-2334-10-187)
Supplement: Additional file 3 — Formulation of the spatial regression model. This file explains how we constructed the semivariogram in the spatial regression model, provides a mathematical formulation of the model, and explains how we fitted the model. [file 1471-2334-10-187-S3.PDF]

Mapping the risk of avian influenza in wild birds in the US: Additional file 3 - Formulation of the spatial regression model

Construction of the semivariogram

Initially, we constructed a generalized linear model with no semivariogram (hereafter the "aspatial model"). Following established protocols, to assess whether it would be appropriate to include a semivariogram into the model, we plotted the semivariogram of the Pearson residuals of the aspatial model Schabenberger and Gotway (2005). The residuals showed spatial structure, so we extended the model to incorporate spatial correlation via a semivariogram. During exploratory data analysis, we tested Gaussian, exponential, and spherical semivariograms. Results indicated that the Gaussian semivariogram provided the best fit to the data.

Notation for the spatial regression model

s_i	county i, $1 \leq i \leq 136$
$\vec{s}$	=vec$(s_1, \ldots, s_{136})$, vector of all of the counties
$x_j(i)$	j^{th} environmental variable calculated on county i, $1 \leq j \leq 12$
$\vec{x_i}$	$= \text{vec}(x_1(i), \ldots, x_{12}(i))$
$m(s_i)$	number of samples tested at county i
$Z(s_i)$	number of AIV-positive samples at county i
$\ln[\lambda(s_i)]$	=$\vec{x_i}\vec{\beta}$
$\mu(s_i)$	$= \lambda(s_i)m(s_i)$
$S(s_i)$	spatial autocorrelation function
h	"lag": The Euclidean distance $\|s_i - s_j\|$, between the centroid of county s_i and the centroid of county s_j, $s_i \neq s_j$
α	"practical range": lag s.t. if $\|s_i - s_j\| \geq \alpha$, then $\rho(Z(s_i), Z(s_j)) \leq 0.05$
$Po(\mu)$	Poisson distribution with mean μ
$G(\mu, \sigma^2)$	Gaussian distribution with mean μ and standard deviation σ

Model formulation

$$Z(s_i)|S(s_i) \sim Po(\mu(s_i)) \tag{1}$$

$$\ln[\mu(s_i)] = \ln[m(s_i)] + \vec{x_i}\vec{\beta} + S(s_i) \tag{2}$$

$$\vec{S}(\vec{s}) \sim G(0, \sigma_S^2 R(\vec{s}, h)) \tag{3}$$

$$R(\vec{s}, h) = C(\vec{s}, h)/\sqrt{Var(\vec{s}) \cdot Var(\vec{s} + h)} \tag{4}$$

$$C(\vec{s}, h) = C(h) = \sigma_S^2 \cdot \exp\left\{-3h^2/\alpha^2\right\} \tag{5}$$

Model fitting

The parameters of the model were estimated via residual pseudo-likelihood using the GLIMMIX procedure in SAS 9.2 to estimate the β coefficients and their standard errors. These coefficients represent the effect of the environmental variables on AIV cases (see Table 1 of the main text).

References

Schabenberger, O., Gotway, C. A., 2005. Statistical Methods for Spatial Data Analysis. Chapman & Hall/CRC, Boca Raton, Florida, USA.
