# Supplementary material for: Mapping the risk of avian influenza in wild birds in the US
Source: BMC Infect Dis. 2010 Jun 23;10:187. doi: 10.1186/1471-2334-10-187 (PMC2912310; doi:10.1186/1471-2334-10-187)

Mapping the Risk of Avian Influenza in Wild Birds in the US

Additional File 4 – Geographic locations of AIV-positive samples in the contiguous US (*n* = 325)

Each sample from BioHealthBase, LaMNA, MAPS, and MAWS has a longitude and latitude. The positive samples from these sources are displayed as red points (*n* = 257). Data from the USDA were assigned to counties but had no longitude and latitude. The positive samples from USDA (*n* = 68) are displayed as green polygons that represent the area of the county where the sample was collected. For the regression model described in the main text, we aggregated all of the data to the county resolution.

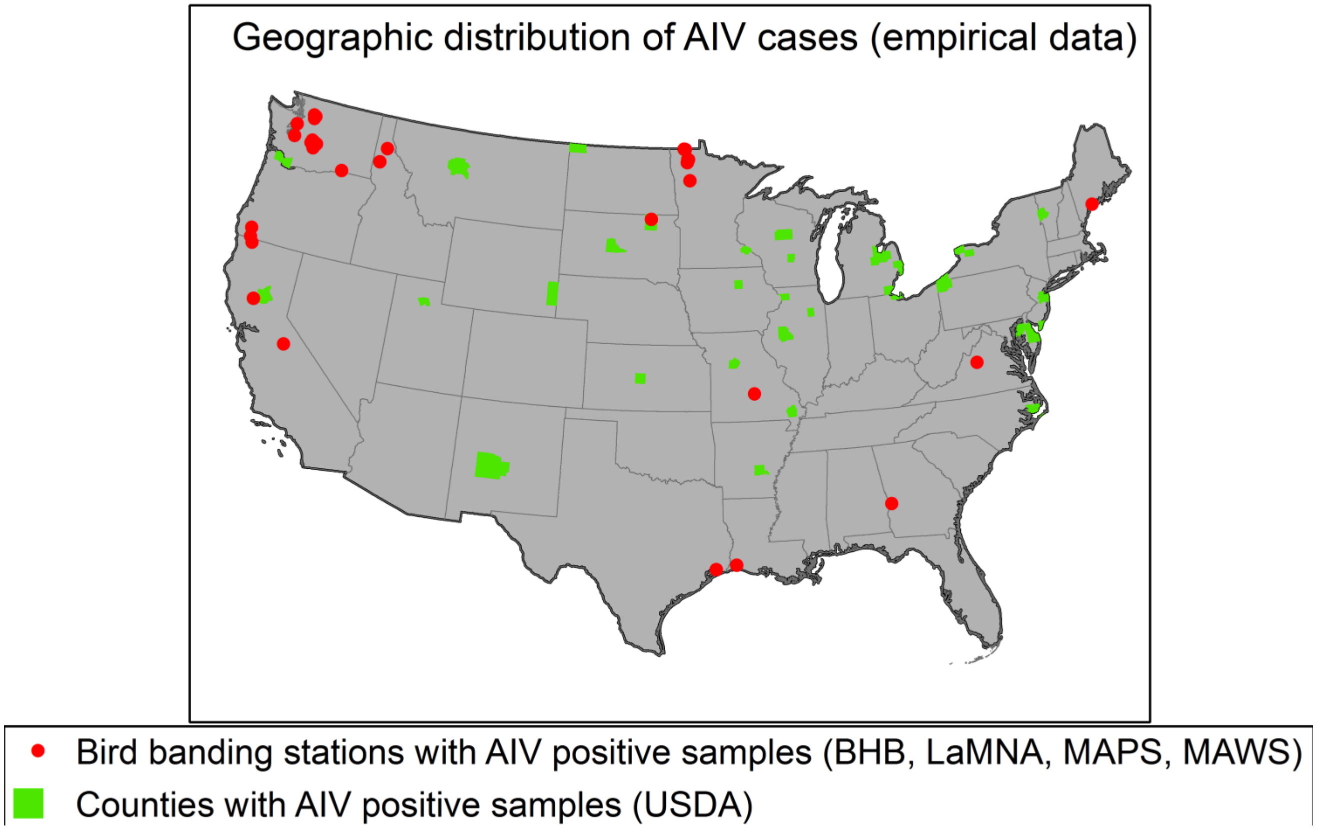

Supplement: Additional file 4 — Geographic locations of AIV-positive samples in the contiguous US (n = 325). This file contains a map showing the bird banding stations where wild birds tested positive for AIV. [file 1471-2334-10-187-S4.PDF]
