# Supplementary material for: Mapping the risk of avian influenza in wild birds in the US
Source: BMC Infect Dis. 2010 Jun 23;10:187. doi: 10.1186/1471-2334-10-187 (PMC2912310; doi:10.1186/1471-2334-10-187)
Supplement: Additional file 5 — Probability of AIV occurrence in US counties or county equivalents. This file consists of a map constructed by modifying the spatial model to generate probabilistic predictions about the influenza in wild birds, which are restricted to being between zero and one, rather than estimates of the number of influenza cases, which range from zero cases to 76 cases per county. [file 1471-2334-10-187-S5.PDF]

Mapping the Risk of Avian Influenza in Wild Birds in the US

Additional File 5 – Probability of AIV occurrence in US counties or county equivalents

The model was constructed by modifying the regression model in the Appendix so that the distribution of the response variable was binomial rather than Poisson [for details, see 1].

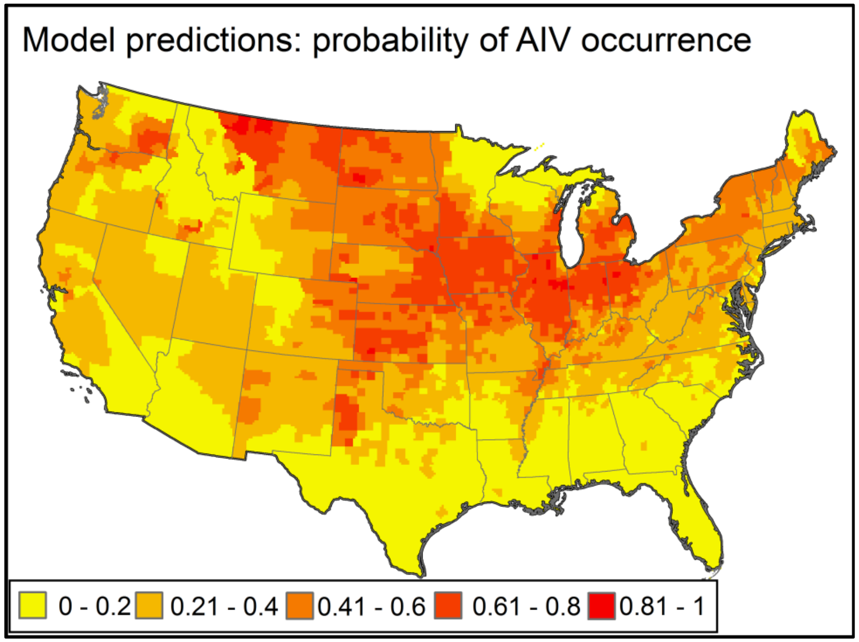

Reference

1. Schabenberger O, Gotway CA: **Statistical Methods for Spatial Data Analysis**. Boca Raton: Chapman & Hall/CRC; 2005.
