# Supplementary material for: Mapping the risk of avian influenza in wild birds in the US
Source: BMC Infect Dis. 2010 Jun 23;10:187. doi: 10.1186/1471-2334-10-187 (PMC2912310; doi:10.1186/1471-2334-10-187)

Mapping the Risk of Avian Influenza in Wild Birds in the US

Additional File 6 – Hotspots of swine production and AIV cases in wild birds in the contiguous US

Data on swine production were obtained from the National Agricultural Statistics Service, US Department of Agriculture. This data set consisted of the number of head of hogs and pigs per US state in 2006-2008, which was the time period of our surveillance of AIV in wild birds. We identified US states in which (i) swine production was high and (ii) we predicted a high number of cases of AIV in wild birds using the model in Additional File 3. Results indicate that the Pacific Northwest is an important AIV hotspot for passerine birds but not a major center for the rearing of swine in the US. In contrast, the Mississippi Flyway in the central US has significant swine production along with a significant number of cases of AIV in ducks. The risk of avian and swine influenza viruses reassorting appears to be greatest at farms in the Mississippi Flyway in the states of Illinois, Iowa, and Minnesota, which are in the top 10% of US states with respect to swine production and are also predicted to be in the top 10% in terms of cases of influenza in wild birds.

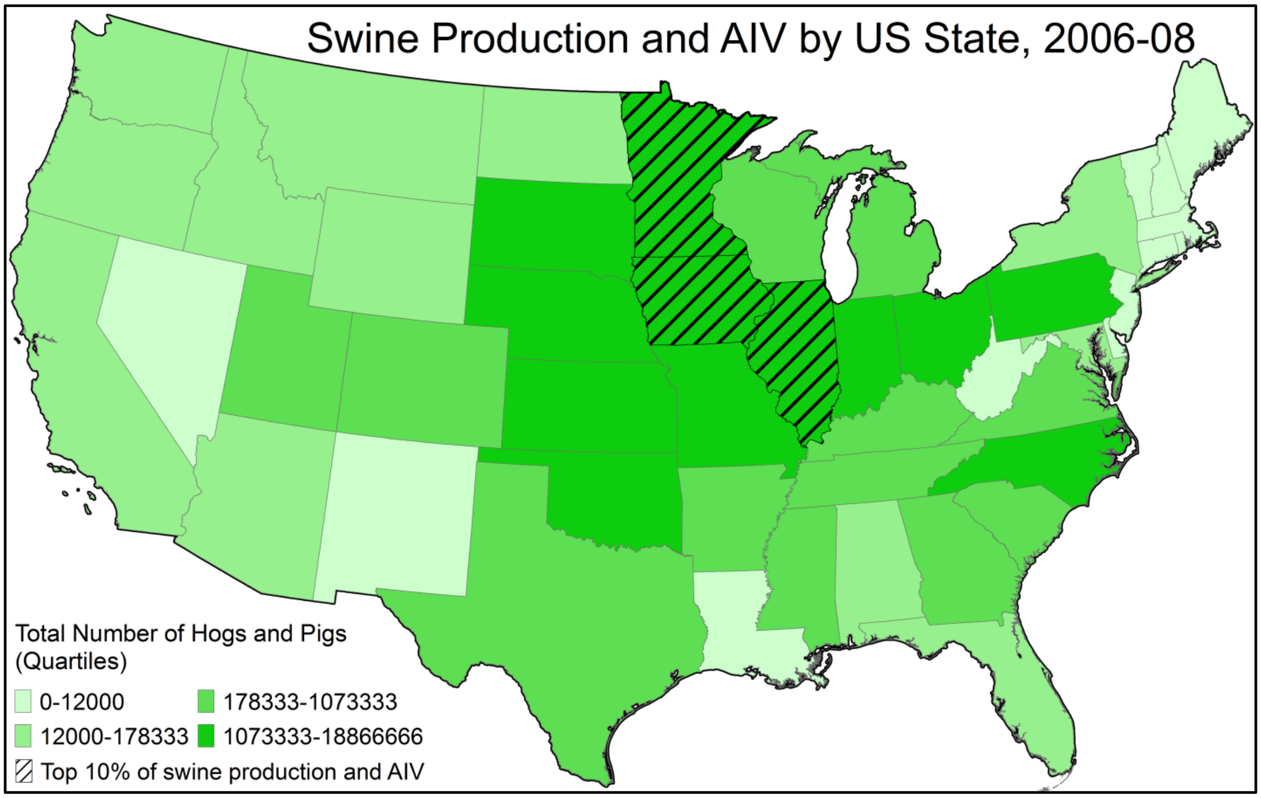

Supplement: Additional File 6 — Hotspots of swine production and AIV cases in wild birds in the contiguous US. This file shows the overlap between areas with intensive swine production in the US and areas in which we predict high prevalence of AIV in wild birds. Reassortment between avian and swine influenza viruses may be more common in such areas. [file 1471-2334-10-187-S6.PDF]
